# Machine Learning algorithm unveils glutamatergic alterations in the *post-mortem* schizophrenia brain

**DOI:** 10.1038/s41537-022-00231-1

**Published:** 2022-02-25

**Authors:** Arianna De Rosa, Andrea Fontana, Tommaso Nuzzo, Martina Garofalo, Anna Di Maio, Daniela Punzo, Massimiliano Copetti, Alessandro Bertolino, Francesco Errico, Antonio Rampino, Andrea de Bartolomeis, Alessandro Usiello

**Affiliations:** 1 CEINGE Biotecnologie Avanzate, 80145 Naples, Italy; 2 Dipartimento di Scienze e Tecnologie Ambientali Biologiche e Farmaceutiche, Università degli Studi della Campania “Luigi Vanvitelli”, 81100 Caserta, Italy; 3 Unit of Biostatistics, Fondazione IRCCS “Casa Sollievo della Sofferenza”, 71013 San Giovanni Rotondo, Italy; 4 Group of Psychiatric Neuroscience, Department of Basic Medical Sciences, Neuroscience and Sense Organs, University of Bari Aldo Moro, 70124 Bari, Italy; 5 Azienda Ospedaliero-Universitaria Policlinico di Bari, 70124 Bari, Italy; 6 Department of Agricultural Sciences, University of Naples “Federico II”, 80055 Portici, Italy; 7 Section of Psychiatry Laboratory of Molecular and Translational Psychiatry, Department of Neuroscience, Reproductive Science and Odontostomatology, School of Medicine, University “Federico II”, 80131 Naples, Italy; 8 Present Address: Department of Biological Chemistry, Center for Epigenetics and Metabolism, U1233 INSERM, University of California, Irvine, CA 92697 USA

**Keywords:** Schizophrenia, Schizophrenia

## Abstract

Schizophrenia is a disorder of synaptic plasticity and aberrant connectivity in which a major dysfunction in glutamate synapse has been suggested. However, a multi-level approach tackling diverse clusters of interacting molecules of the glutamate signaling in schizophrenia is still lacking. We investigated in the post-mortem dorsolateral prefrontal cortex (DLPFC) and hippocampus of schizophrenia patients and non-psychiatric controls, the levels of neuroactive d- and l-amino acids (l-glutamate, d-serine, glycine, l-aspartate, d-aspartate) by HPLC. Moreover, by quantitative RT-PCR and western blotting we analyzed, respectively, the mRNA and protein levels of pre- and post-synaptic key molecules involved in the glutamatergic synapse functioning, including glutamate receptors (NMDA, AMPA, metabotropic), their interacting scaffolding proteins (PSD-95, Homer1b/c), plasma membrane and vesicular glutamate transporters (EAAT1, EAAT2, VGluT1, VGluT2), enzymes involved either in glutamate-dependent GABA neurotransmitter synthesis (GAD65 and 67), or in post-synaptic NMDA receptor-mediated signaling (CAMKIIα) and the pre-synaptic marker Synapsin-1. Univariable analyses revealed that none of the investigated molecules was differently represented in the post-mortem DLPFC and hippocampus of schizophrenia patients, compared with controls. Nonetheless, multivariable hypothesis-driven analyses revealed that the presence of schizophrenia was significantly affected by variations in neuroactive amino acid levels and glutamate-related synaptic elements. Furthermore, a Machine Learning hypothesis-free unveiled other discriminative clusters of molecules, one in the DLPFC and another in the hippocampus. Overall, while confirming a key role of glutamatergic synapse in the molecular pathophysiology of schizophrenia, we reported molecular signatures encompassing elements of the glutamate synapse able to discriminate patients with schizophrenia and normal individuals.

## Introduction

Schizophrenia has been conceptualized as a disease of dysfunctional synaptic plasticity^1^ and aberrant cortical–subcortical connectivity^2,3^, with a multigenic etiopathogenesis and a complex biological architecture, which likely involves different biological pathways^4,5^. Genome Wide Association Studies (GWASs) have confirmed that a large pool of genetic variants are associated with the disorder and that genetic risk identified by such variants converges onto a relatively small number of biologically meaningful trajectories or pathways^6^. Interestingly, about 30% of genomic variation associated with schizophrenia by GWASs is directly or indirectly related with the glutamatergic signaling in the central nervous system, confirming the hypothesis that alteration of the glutamate synapse may play a critical role in the pathophysiology of this disorder (glutamatergic hypothesis of schizophrenia)^5^. Consistently, alteration in the overall glutamatergic synapse composition, which includes glutamatergic NMDA, AMPA and metabotropic receptors (NMDARs, AMPARs, and mGluRs, respectively), along with scaffolding and adaptor proteins^7–10^, as well as membrane and vesicular transporters^11–14^ may occur in the brain of schizophrenia patients. In line with such evidence, genomic studies have reported that glutamate-pathway-specific polygenic risk scores predict behavioral and neuroimaging endophenotypes of schizophrenia supported by dysfunctions of the hippocampus and prefrontal cortex (PFC), two brain regions of critical importance to the pathophysiology of schizophrenia^15,16^. In particular, it has been suggested that dysregulation of the PFC in schizophrenia is secondary to an early alteration of the glutamatergic neurotransmission in the hippocampus^17^. However, to what extent each element of glutamate neurotransmission contributes to the appearance of clinical phenotypes of schizophrenia still remains obscure. Similarly, it stands unclear whether variation of one single element, rather than the coordinated variation of levels of many elements of the glutamatergic synapse is critical to the onset of the disorder.

To shed new light on such cryptic areas of our understanding for schizophrenia, here we performed a multimodal research of post-mortem dorsolateral PFC (DLPFC) and hippocampus of schizophrenia patients and non-psychiatric individuals on which we used high-performance liquid chromatography (HPLC) analysis to measure levels of glutamate and related amino acids with neurotransmitter/neuromodulatory activity, including d-amino acids, whose role as endogenous NMDAR modulators is emerging significantly in schizophrenia pathophysiology^18,19^. Moreover, in the same brain areas, by quantitative RT-PCR (qRT-PCR) and western blotting analyses we investigated, respectively, mRNA and protein levels of pre- and post-synaptic key molecules, including glutamate receptors (NMDAR, AMPAR, metabotropic), their interacting scaffolding proteins (PSD-95, Homer1b/c), plasma membrane and vesicular glutamate transporters (EAAT1, EAAT2, VGluT1, VGluT2), enzymes involved either in glutamate-dependent GABA neurotransmitter synthesis (GAD65 and 67), or in post-synaptic NMDAR-signaling, such as CAMKIIα and the pre-synaptic marker Synapsin-1^20,21^. We used a multistep approach to analyze data: (1) a univariable statistical analysis to detect possible significant differences between patients and controls in the levels of molecular and neurochemical elements previously mentioned; (2) a hypothesis-driven multivariable statistical analysis, and (3) a hypothesis-free Machine Learning analysis. The latter allowed us to (a) detect pathways of molecules able to discriminate schizophrenia cases from controls on the basis of the joint distribution of their levels in the DLPFC and the hippocampus; (b) identify interactions among elements of the synapse that defined molecular signatures of schizophrenia cases and controls.

## Results

### HPLC analysis of neuroactive d- and l-amino acids levels in the post-mortem DLPFC and hippocampus of schizophrenia and control subjects

**Fig. 1:**
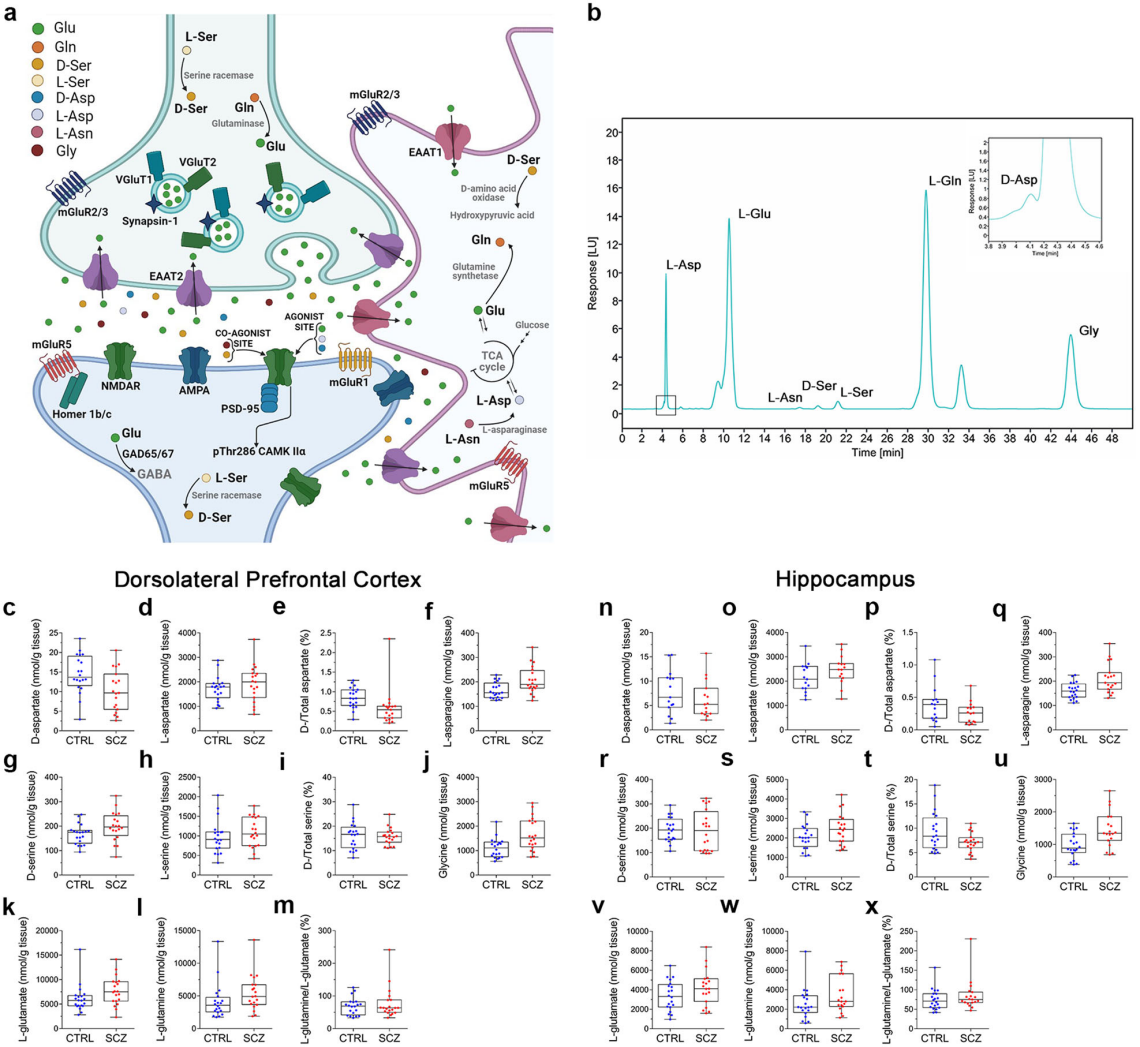
Analysis of d-aspartate, l-aspartate, d-serine, l-serine, l-asparagine, l-glutamate, l-glutamine and glycine levels in the post-mortem dorsolateral prefrontal cortex and hippocampus of schizophrenia patients and control subjects. **a** Schematic model of the tripartite glutamatergic synapse showing the main localization of the molecules analyzed in this study. Image created with BioRender.com (www.biorender.com). **b** Representative HPLC chromatogram showing d-aspartate (d-Asp), l-aspartate (l-Asp), l-glutamate (l-Glu), l-asparagine (l-Asn), d-serine (d-Ser), l-serine (l-Ser), l-Glutamine (l-Gln) and glycine (Gly) peaks obtained from non-psychiatric human DLPFC homogenate. **c**–**x** Content of **c**, **n** d-aspartate, **d**, **o** l-aspartate, **e**, **p** d-aspartate/total aspartate ratio, **f**, **q** l-asparagine, **g**, **r** d-serine, **h**, **s** l-serine, **i**, **t** d-serine/total serine ratio, **j**, **u** glycine, **k**, **v** l-glutamate, **l**, **w** l-glutamine levels and **m**, **x** l-glutamine/l-glutamate evaluated in the post-mortem **c**–**m** dorsolateral prefrontal cortex (DLPFC) and **n**–**x** hippocampus, compared between controls (CTRL) and patients with schizophrenia (SCZ). In each sample, all the amino acids were detected in a single run by HPLC and expressed as nmol/g of tissue, while the ratios are expressed as percentage (%). The number of examined samples is reported in Table 2, for each considered amino acid.

Compelling evidence supports the hypothesis that deficient glutamatergic activity contributes in schizophrenia etiology and pathophysiology^22^. Accordingly, deregulation of glutamate and neuroactive d-amino acids levels has been reported in schizophrenia brains^5,23,24^. Here, we measured by HPLC the levels of the amino acids l-Glu, l-Asp, d-Asp, d-Ser, Gly, known to stimulate and modulate the activity of NMDARs^25–27^, and their precursors, l-Gln, l-Asn, and l-Ser in the DLPFC and hippocampus of schizophrenia patients and non-psychiatric controls (*n* = 20/brain region/clinical condition). Before proceeding with statistical comparisons of amino acids levels between schizophrenia and controls, we assessed whether the two groups were imbalanced with respect to the following clinical variables (confounders): gender, age at deceased, post-mortem interval (PMI) and samples’ pH. No statistically significant differences were found in gender (number of males (%): CTRL = 16 (80%), SCZ = 12 (60%), *χ*^2^ = 1.071, df=1, *p* = 0.301 from Chi-Square test) and pH (median [IQR]: CTRL = 6.54 [6.49–6.63], SCZ = 6.50 [6.42–6.56], *t* = 0.708, df = 26, *p* = 0.485 from two-sample *t* test on log values), while significant differences were found in age (median [IQR]: CTRL = 73.5 [66.0–80.3] years, SCZ = 52.5 [39.5–61.3] years, *t* = 4.819, df = 38, *p* < 0.001 from two-sample *t* test) and PMI (median [IQR]: CTRL = 12.9 [11.8–16.3] h, SCZ = 15.3 [12.5–24.6] h, *t* = −2.426, df = 38, *p* = 0.020 from two-sample *t* test on log values) (Table 1 and Supplementary Table 3 for individual characteristics). On the basis of these findings, we assessed the differences between groups in each of the molecule reported above using ANCOVA models, including both age and PMI as confounders. In the DLPFC, this analysis revealed significantly higher levels in l-Asn and Gly in schizophrenia patients, compared to controls (adjusted means [95% CI] for l-Asn: CTRL = 159.2 [135.2–183.3], SCZ = 213.2 [189.2–237.3] nmol/g tissue, *F*(1,36) = 8.167, *p* = 0.007; Gly: CTRL = 1103.8 [813.4–1394.3], SCZ = 1652.1 [1361.6–1942.5] nmol/g tissue, *F*(1,36) = 5.780, *p* = 0.021; Fig. 1f,j, Table 2). Moreover, we found a borderline significantly higher levels in d-Ser in schizophrenia patients, compared to controls (adjusted means [95% CI]: CTRL = 160.8 [133.5–188.1], SCZ = 204.2 [176.9–231.5] nmol/g tissue, *F*(1,36) = 4.097, *p* = 0.050; Fig. 1g, Table 2). On the other hand, no significant differences were found in d-Asp, l-Asp, l-Ser, l-Glu, and l-Gln levels, as well as in d-Asp/total Asp, d-Ser/total Ser and L-Gln/L-Glu ratios (Fig. 1c-e,h,i,k-m, Table 2). Also in the hippocampus, we found a significantly higher levels in l-Asn in schizophrenia patients, compared to controls (adjusted means [95% CI]: CTRL = 163.1 [137.0–189.2], SCZ = 210.4 [183.5–237.4] nmol/g tissue, *F*(1,35) = 5.169, *p* = 0.029; Fig. 1q), while no alterations in other amino acids were detected (Fig. 1n–p,r–x, Table 2).

**Table 1:** Demographic and clinical characteristics of control subjects and schizophrenia patients.

Characteristics	Control	Schizophrenia	Statistic	*p* value
Subjects (total number)	20	20	–	–
Gender (M/F)	16/4	12/8	*χ*^2^ = 1.071 (df=1)	0.301^a^
Age (years, median [IQR])	73.50 [66.00–80.25]	52.50 [39.50−61.25]	*t* = 4.819 (df = 38)	<0.001^b^
PMI (hours, median [IQR])	12.90 [11.80 −16.32]	15.25 [12.52−24.58]	*t* = −2.426 (df = 38)	0.020^c^
pH (median, [IQR])	6.54 [6.49–6.63]	6.50 [6.42−6.56]	*t* = 0.708 (df = 26)	0.485^c^
RIN (median, [IQR])	6.05 [5.50−7.12]	6.75 [6.05 − 7.08]	*t* = −0.036 (df = 38)	0.971^b^

Continuous variables are reported as median along with IQR.

*M/F* number of males/females, *PMI* post-mortem interval, *RIN* RNA integrity number, *IQR* Interquartile Range (i.e. first-third quartiles), *df* degrees of freedom.

^a^*p*-value from Chi-Square test (with Yates’s correction).

^b^*p*-value from two sample *t*-test.

^c^*p*-value from two sample *t*-test on log transformed values.

**Table 2:** Comparisons of amino acids levels (expressed as nmol/g of tissue, while ratios are expressed as percentages) in the post-mortem dorsolateral prefrontal cortex and hippocampus between patients with schizophrenia and control subjects.

Amino acid	DLPFC						HIP					
	CTRL		SCZ		Statistics		CTRL		SCZ		Statistics	
	Mean (95% CI)	No.	Mean (95% CI)	No.	*F*(d*f*_1_,d*f*_2_); *p*-value raw*	*p*-value adjusted^#^	Mean (95% CI)	No.	Mean (95% CI)	No.	*F*(d*f*_1_,d*f*_2_); *p*-value raw*	*p*-value adjusted^#^
d-aspartate	14.6 (11.3–17.9)	20	11.1 (7.8–14.4)	20	*F*(1,36) = 1.846; *p* = 0.183	1.000	8.2 (5.5–10.8)	15	5.8 (3.2– 8.5)	15	*F*(1,26) = 1.317; *p* = 0.262	1.000
l-aspartate	1647.3 (1321.1–1973.5)	20	2012.4 (1686.2–2338.6)	20	*F*(1,36) = 2.034; *p* = 0.162	1.000	2027.9 (1680.0–2375.7)	15	2572.5 (2224.6–2920.3)	15	*F*(1,26) = 4.101; *p* = 0.053	0.586
d-aspartate/total aspartate	0.8 (0.6–1.1)	20	0.6 (0.4–0.8)	20	*F*(1,36) = 1.631; *p* = 0.210	1.000	0.4 (0.3–0.6)	15	0.2 (0.1–0.4)	15	*F*(1,26) = 3.453; *p* = 0.075	0.820
d-serine	160.8 (133.5–188.1)	20	204.2 (176.9–231.5)	20	*F*(1,36) = 4.097; *p* = 0.050	0.555	199.2 (165.3–233.0)	20	185.2 (151.3–219.1)	20	*F*(1,36) = 0.276; *p* = 0.603	1.000
l-serine	901.6 (690.5–1112.8)	20	1170.8 (959.6–1381.9)	20	*F*(1,36) = 2.637; *p* = 0.113	1.000	2038.2 (1674.4–2402.0)	20	2480.0 (2116.2–2843.8)	20	*F*(1,36) = 2.393; *p* = 0.131	1.000
d-serine/total serine	16.4 (14.0–18.9)	20	15.4 (13.0–17.9)	20	*F*(1,36) = 0.281; *p* = 0.600	1.000	9.6 (7.9–11.2)	20	7.0 (5.3–8.6)	20	*F*(1,36) = 3.964; *p* = 0.054	0.595
l-glutamate	6424.3 (4889.5–7959.2)	20	7374.9 (5840.0–8909.7)	20	*F*(1,36) = 0.622; *p* = 0.435	1.000	3499.5 (2599.9–4399.2)	20	4081.3 (3151.9–5010.7)	19	*F*(1,35) = 0.658; *p* = 0.423	1.000
l-glutamine	3996.3 (2531.3–5461.3)	20	5606.4 (4141.4–7071.4)	20	*F*(1,36) = 1.961; *p* = 0.170	1.000	2625.8 (1700.0–3551.6)	20	3395.5 (2439.1–4351.9)	19	*F*(1,35) = 1.087; *p* = 0.304	1.000
l-glutamine/l-glutamate	63.7 (43.1–84.3)	20	81.8 (61.2–102.4)	20	*F*(1,36) = 1.247; *p* = 0.271	1.000	72.3 (54.5–90.2)	20	87.7 (69.3–106.2)	19	*F*(1,35) = 1.174; *p* = 0.286	1.000
l-asparagine	159.2 (135.2–183.3)	20	213.2 (189.2–237.3)	20	*F*(1,36) = 8.167; *p* = 0.007	0.078	163.1 (137.0–189.2)	20	210.4 (183.5–237.4)	19	*F*(1,35) = 5.169; *p* = 0.029	0.322
Glycine	1103.8 (813.4–1394.3)	20	1652.1 (1361.6–1942.5)	20	*F*(1,36) = 5.780; *p* = 0.021	0.236	1034.7 (783.9–1285.5)	20	1387.1 (1127.9–1646.2)	19	*F*(1,35) = 3.104; *p* = 0.087	0.955

All the amino acids were detected in a single run by HPLC. Results are reported as age and post-mortem interval adjusted means along with their 95% confidence interval (CI).

*DLPFC* dorsolateral prefrontal cortex, *HIP* hippocampus, *CTRL* control subjects, *SCZ* patients with schizophrenia, No. = number of subjects with non-missing values for each considered variable.

*To test the difference of adjusted means between the two groups, *p*-values (raw) were computed from ANCOVA models which include the presence of SCZ as the main grouping variable and age and post-mortem interval as confounders.

^#^Adjusted *p*-values correspond to the raw *p*-values corrected for multiple testing following the Bonferroni method. *F*(d*f*_1_,d*f*_2_) is the quantile of the *F*-distribution with d*f*_1_ and d*f*_2_ degrees of freedom corresponding to main grouping variable effect.

However, after the correction of *p* values for multiple testing, no statistically significant differences were found in any of the molecules analyzed in both brain regions between schizophrenia and non-psychiatric subjects (Fig. 1, Table 2).

### Analysis of the expression of glutamatergic synapse-related genes and proteins in the post-mortem DLPFC and hippocampus of schizophrenia and control subjects

GWASs have identified several genes encoding proteins implicated in glutamatergic functioning as risk genes for schizophrenia^28,29^. Based on these findings, we evaluated the mRNA and protein expression of genes implicated in glutamatergic signaling at both pre-synaptic and post-synaptic level in the post-mortem DLPFC and hippocampus of the same schizophrenia and non-psychiatric subjects analyzed for amino acids content. Through qRT-PCR and western blotting we analyzed, respectively, the mRNA (name indicated below in brackets) and protein levels of the subunits of the NMDARs, GluN1 (*GRIN1*), GluN2A (*GRIN2A*), GluN2B (*GRIN2B*), and of the AMPARs, GluA1 (*GRIA1*), GluA2/3 (*GRIA2/3*) and GluA4 (*GRIA4*), the metabotropic glutamate receptors, mGluR1 (*GRM1*), mGluR2/3 (*GRM2, GRM3*) and mGluR5 (*GRM5*), the post-synaptic density proteins, Homer1b/c (*Homer1*) and PSD-95 (*DLG4*), the glutamate decarboxylase isoforms, GAD65 (*GAD1*) and GAD67 (*GAD2*), the excitatory amino acids transporters, EAAT1 (*SLC1A3*) and EAAT2 (*SLC1A2*), the vesicular glutamate transporters, VGluT1 (*SLC17A7*) and VGluT2 (*SLC17A6*), the synaptic vesicle membrane protein, Synapsin-1 (*SYN1*), the calcium/calmodulin-dependent protein kinase II alpha, CaMKIIα (*CAMK2A*), and its phosphorylated form, Thr286-P-CaMKIIα.

**Fig. 2:**
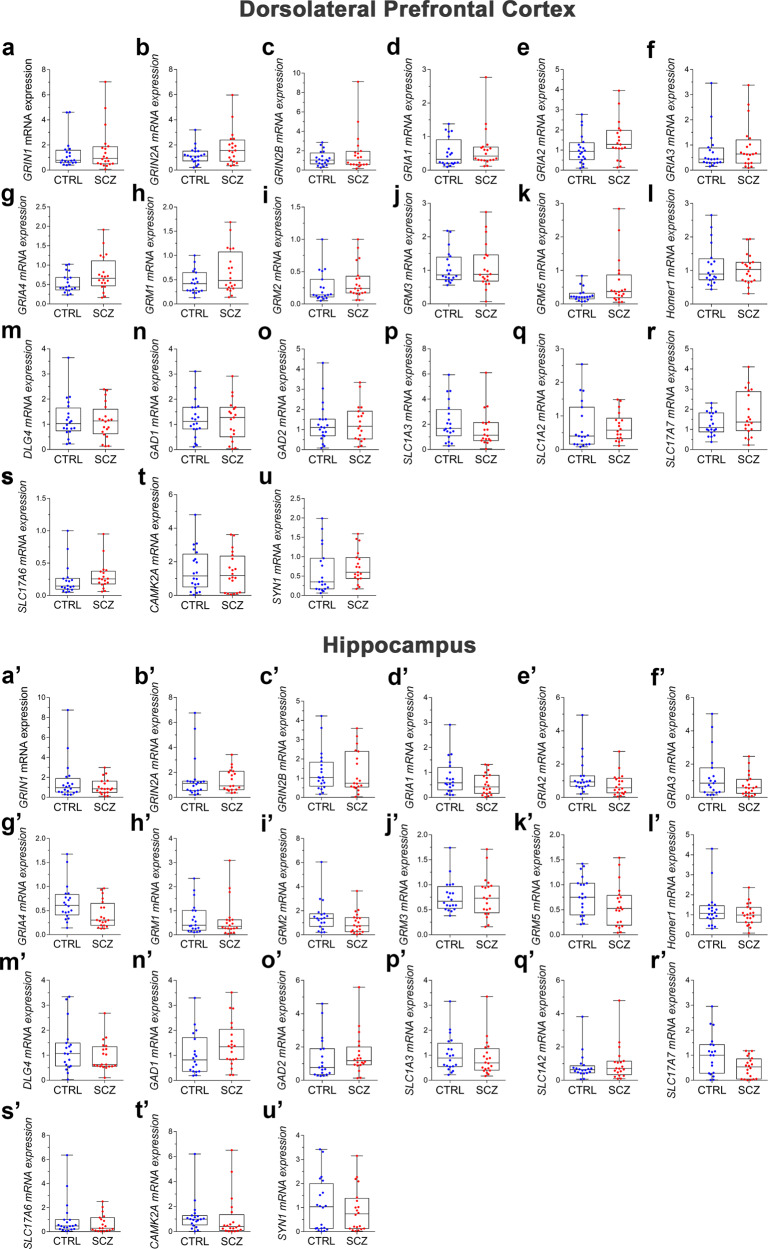
Glutamatergic synapse-related gene expression in the post-mortem dorsolateral prefrontal cortex and hippocampus of control subjects and patients with schizophrenia. mRNA expression levels of **a**, **a**′ *GRIN1*, **b**, **b**′ *GRIN2A*, **c**, **c**′ *GRIN2B*, **d**–**d**′ *GRIA1*, **e,e**′ *GRIA2*, **f**, **f**′ *GRIA3*, **g**, **g**′ *GRIA4*, **h**, **h**′ *GRM1*, **i**, **i**′ *GRM2*, **j**, **j**′ *GRM3*, **k**, **k**′ *GRM5*, **l**, **l**′ *Homer1*, **m**, **m**′ *DLG4*, **n**–**n**′ *GAD1*, **o**, **o**′ *GAD2*, **p**, **p**′ *SLC1A3*, **q**, **q**′ *SLC1A2*, **r**, **r**′ *SLC17A7*, and **s**, **s**’ *SLC17A6,* **t**, **t**′ *CAMK2A,* and **u**, **u**′ *SYN1* in the **a**–**u** dorsolateral prefrontal cortex and **a**′–**u**′ hippocampus homogenates of control (CTRL) and schizophrenia (SCZ) patients. mRNA expression was normalized to the mean of two housekeeping genes, *β-actin* and *cyclophilin* (*PPIA*), and expressed as arbitrary units. The number of examined samples is reported in Supplementary Table 4, for each considered mRNA.

As regards to transcripts, we found that *GRIA2* and *Homer1* levels resulted significantly lower in the hippocampus of schizophrenia patients, compared with non-psychiatric controls from ANCOVA models (adjusted means [95% CI]; *GRIA2*: CTRL = 1.1 [0.7–1.8], SCZ = 0.5 [0.3–0.7] arbitrary units, *F*(1,36) = 6.226, *p* = 0.017; *Homer1*: CTRL = 1.3 [0.9–1.8], SCZ = 0.7 [0.5–0.9] arbitrary units, *F*(1,36) = 6.787, *p* = 0.013; Fig. 2e′, l′, Supplementary Table 4), while no significant alterations were reported in the levels of the other hippocampal and cortical mRNAs detected (Fig. 2, Supplementary Table 4).

**Fig. 3:**
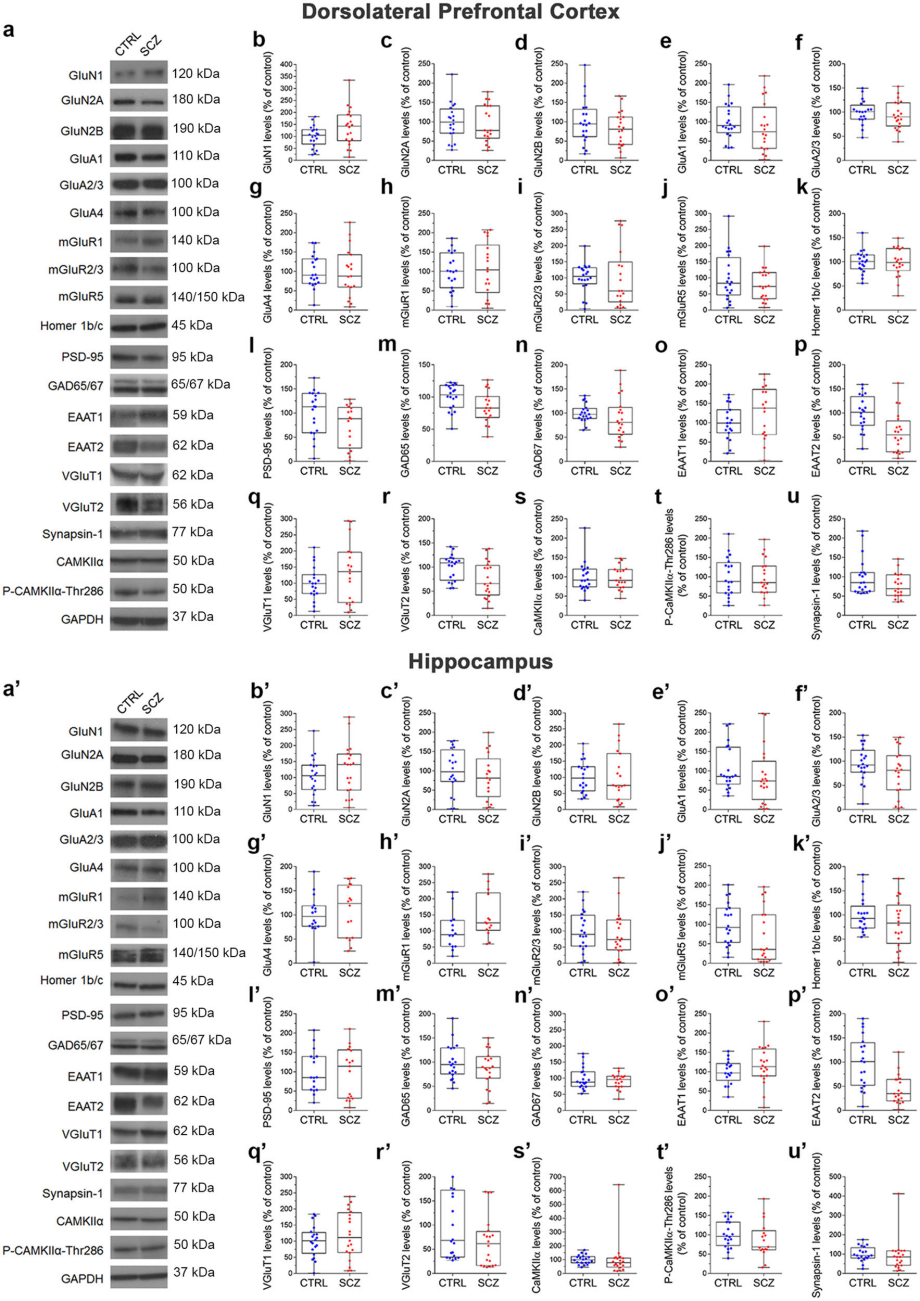
Glutamatergic synapse-related protein expression in the post-mortem dorsolateral prefrontal cortex and hippocampus of control subjects and patients with schizophrenia. **a**, **a**′ Representative autoradiograms of immunoblots of the glutamatergic synapse-related proteins performed in the dorsolateral prefrontal cortex and hippocampus of non-psychiatric controls (CTRL) and patients with schizophrenia (SCZ). Quantification of **b**, **b**′ GluN1, **c**, **c**′ GluN2A, **d**, **d**′ GluN2B, **e**, **e**′ GluA1, **f**, **f**′ GluA2/3, **g**, **g**′ GluA4, **h**, **h**′ mGluR1, **i**, **i**′ mGluR2/3, **j**, **j**′ mGluR5, **k**, **k**’) Homer1b/c, **l**, **l**′ PSD-95, **m**, **m**′ GAD65, **n**, **n**′ GAD67, **o**, **o**′ EAAT1, **p**′, **p**′ EAAT2, **q**, **q**′ VGluT1, **r**, **r**′ VGluT2, **s**, **s**′ CaMKIIα, **t**, **t**′ Thr286-P-CaMKIIα and **u**, **u**’ Synapsin-1 protein levels in the **a**–**u** dorsolateral prefrontal cortex and **a**′–**u**′ hippocampus of control subjects and patients with schizophrenia. GAPDH was used to normalize for variations in loading and transfer. The number of examined samples is reported in Supplementary Table 5, for each considered protein. Raw blots are shown in the Supplementary Fig. 1 (dorsolateral prefrontal cortex) and Supplementary Fig. 2 (hippocampus).

As regards to proteins, we observed a significantly higher levels in EAAT1, and lower levels in GluN2A and EAAT2 in the DLPFC of schizophrenia patients, compared with non-psychiatric controls (adjusted means [95% CI]; EAAT1: CTRL = 87.8 [59.2–116.3], SCZ = 140.4 [109.7–171.0]% of control, *F*(1,32) = 5.123, *p* = 0.031; GluN2A: CTRL = 117.3 [96.2–138.4], SCZ = 76.0 [54.2–97.8]% of control, *F*(1,35) = 5.965, *p* = 0.020; EAAT2: CTRL = 97.0 [76.4–117.5], SCZ = 62.6 [42.0–83.2]% of control, *F*(1,36) = 4.524, *p* = 0.04; Fig. 3o,c,p, Supplementary Fig. 1, Supplementary Table 5). EAAT2 levels were significantly lower also in the hippocampus of schizophrenia patients, while a significantly higher levels in mGluR1 were found in this brain region, compared to controls (adjusted means [95% CI]; EAAT2: CTRL = 97.1 [73.3–120.8], SCZ = 46.4 [21.8–70.9]% of control, *F*(1,35) = 7.078, *p* = 0.012; mGluR1: CTRL = 88.1 [49.0–127.3], SCZ = 158.4 [119.3–197.5]% of control, F(1,24) = 5.435, *p* = 0.028; (Fig. 3p′,h′, Supplementary Fig. 2, Supplementary Table 5). All other cortical and hippocampal proteins were comparable between diagnoses (Fig. 3, Supplementary Figs. 1, 2, Supplementary Table 5).

However, also in this case, after the correction of *p* values for multiple testing, no statistically significant differences were found in any of the analyzed transcripts or proteins between schizophrenia and non-psychiatric subjects in both brain regions (Figs. 2, 3, Supplementary Tables 4, 5).

### Linear combinations of multiple molecules of the glutamatergic synaptic components are predictive of schizophrenia in the post-mortem DLPFC

Here, we generated arbitrary (hypothesis-driven) multivariable logistic models, defined on the basis of the functional interaction among different neurochemical and molecular elements of the glutamatergic synapse and tested whether linear combination of these molecules could discriminate between control and schizophrenia group, in both the DLPFC and hippocampus (Table 3, Supplementary Table 6).

**Table 3:** Results from multivariable logistic regression models which include a linear combination of multiple molecules of synaptic components (chosen a priori) as main covariates along with age and post-mortem interval (PMI) as confounders.

Cluster	DLPFC					HIP				
	N.SCZ/total	Variable	OR (95% CI)	*p*-value	Deviance test statistic; *p*-value^*^	N.SCZ/total	Variable	OR (95% CI)	*p*-value	Deviance test statistic; *p*-value^*^
GluN1 + d-serine	20/40	Age	0.84 (0.71–0.93)	0.006	*χ*^2^ = 8.515 (df = 2); *p* = 0.014	20/40	Age	0.86 (0.74–0.93)	0.005	*χ*^2^ = 2.764 (df = 2); *p* = 0.251
		PMI	1.35 (1.08–1.88)	0.025			PMI	1.35 (1.06–1.89)	0.034	
		GluN1	1.00 (0.99–1.02)	0.543			GluN1	1.02 (1.00–1.04)	0.142	
		d-serine	1.03 (1.01–1.07)	0.028			d-serine	1.00 (0.98–1.02)	0.839	
l-glutamate + mGluR2/3 + mGluR5 + EAAT2	19/39	Age	0.83 (0.65–0.93)	0.019	*χ*^2^ = 11.065 (df = 4); *p* = 0.026	18/38	Age	0.89 (0.79–0.97)	0.021	*χ*^2^ = 6.857 (df = 4); *p* = 0.144
		PMI	1.25 (0.94–2.02)	0.212			PMI	1.22 (0.98–1.69)	0.138	
		l-glutamate	1.00 (1.00–1.00)	0.080			l-glutamate	1.00 (1.00–1.00)	0.335	
		mGluR2/3	1.01 (0.99–1.05)	0.224			mGluR2/3	0.99 (0.97–1.01)	0.494	
		mGluR5	0.97 (0.92–1.00)	0.087			mGluR5	1.00 (0.99–1.02)	0.718	
		EAAT2	0.95 (0.89–0.99)	0.075			EAAT2	0.97 (0.93–1.00)	0.049	
GluA1 + PSD-95	17/35	Age	0.77 (0.57–0.90)	0.013	*χ*^2^ = 7.945 (df = 2); *p* = 0.019	17/34	Age	0.87 (0.75–0.94)	0.008	*χ*^2^ = 0.404 (df = 2); *p* = 0.817
		PMI	1.29 (0.96–2.05)	0.147			PMI	1.24 (1.01–1.65)	0.069	
		GluA1	0.96 (0.91–1.00)	0.112			GluA1	1.00 (0.98–1.01)	0.586	
		PSD-95	0.97 (0.92–1.01)	0.196			PSD-95	1.00 (0.98–1.02)	0.747	
l-glutamate + GluN2A + GluN2B	19/39	Age	0.78 (0.59–0.90)	0.017	*χ*^2^ = 7.781 (df = 3); *p* = 0.051	16/35	Age	0.86 (0.73–0.94)	0.013	*χ*^2^ = 4.252 (df = 3); *p* = 0.236
		PMI	1.31 (1.02–1.87)	0.061			PMI	1.24 (0.97–1.78)	0.161	
		l-glutamate	1.00 (1.00–1.00)	0.210			l-glutamate	1.00 (1.00–1.00)	0.413	
		GluN2A	0.95 (0.90–0.99)	0.058			GluN2A	0.98 (0.94–1.00)	0.136	
		GluN2B	1.02 (0.99–1.07)	0.244			GluN2B	1.02 (1.00–1.06)	0.073	
GluN2A + GluN2B	19/39	Age	0.81 (0.66–0.91)	0.008	*χ*^2^ = 5.847 (df = 2); *p* = 0.054	17/36	Age	0.85 (0.72–0.93)	0.010	*χ*^2^ = 3.473 (df = 2); *p* = 0.176
		PMI	1.26 (1.01–1.73)	0.076			PMI	1.19 (0.96–1.58)	0.152	
		GluN2A	0.96 (0.92–0.99)	0.050			GluN2A	0.98 (0.95–1.01)	0.175	
		GluN2B	1.02 (0.99–1.05)	0.233			GluN2B	1.02 (1.00–1.05)	0.093	
PSD95 + GluN2A + GluN2B	16/34	Age	0.75 (0.51–0.89)	0.022	*χ*^2^ = 7.327 (df = 3); *p* = 0.062	14/31	Age	0.84 (0.69–0.93)	0.015	*χ*^2^ = 2.289 (df = 3); *p* = 0.515
		PMI	1.44 (1.05–2.73)	0.083			PMI	1.24 (0.99–1.71)	0.105	
		PSD-95	0.98 (0.93–1.02)	0.296			PSD-95	0.99 (0.97–1.02)	0.570	
		GluN2A	0.95 (0.87–1.00)	0.117			GluN2A	0.99 (0.95–1.02)	0.396	
		GluN2B	1.03 (0.97–1.09)	0.299			GluN2B	1.02 (0.99–1.06)	0.149	
Synapsin-1 + VGluT1 + VGluT2	17/36	Age	0.83 (0.65–0.93)	0.020	*χ*^2^ = 7.030 (df = 3); *p* = 0.071	20/40	Age	0.89 (0.80–0.95)	0.004	*χ*^2^ = 0.913 (df = 3); *p* = 0.822
		PMI	1.19 (0.88–1.79)	0.295			PMI	1.20 (0.99–1.54)	0.098	
		Synapsin-1	0.97 (0.91–1.01)	0.350			Synapsin-1	1.00 (0.99–1.02)	0.908	
		VGluT1	1.02 (0.99–1.07)	0.266			VGluT1	1.00 (0.99–1.02)	0.625	
		VGluT2	0.97 (0.91–1.01)	0.156			VGluT2	0.99 (0.98–1.01)	0.392	
GluN1 + Glycine	20/40	Age	0.88 (0.79–0.95)	0.009	*χ*^2^ = 4.859 (df = 2); *p* = 0.088	19/39	Age	0.87 (0.76–0.96)	0.017	*χ*^2^ = 5.134 (df = 2); *p* = 0.077
		PMI	1.26 (1.04–1.64)	0.038			PMI	1.40 (1.08–2.11)	0.038	
		GluN1	1.00 (0.98–1.01)	0.690			GluN1	1.02 (1.00–1.04)	0.132	
		Glycine	1.00 (1.00–1.00)	0.071			Glycine	1.00 (1.00–1.00)	0.154	
EAAT2 + PSD-95 + Homer 1b/c	17/35	Age	0.83 (0.67–0.93)	0.013	*χ*^2^ = 6.697 (df = 3); *p* = 0.082	16/33	Age	0.85 (0.67–0.95)	0.039	*χ*^2^ = 5.766 (df = 3); *p* = 0.124
		PMI	1.46 (1.08–2.44)	0.053			PMI	1.34 (1.03–2.16)	0.092	
		EAAT2	0.97 (0.92–1.01)	0.190			EAAT2	0.96 (0.92–0.99)	0.053	
		PSD-95	0.97 (0.91–1.01)	0.164			PSD-95	1.00 (0.97–1.02)	0.848	
		Homer 1b/c	0.99 (0.94-1.04)	0.677			Homer 1b/c	1.03 (0.99–1.10)	0.249	
GluN2A + CAMKIIα	18/38	Age	0.81 (0.66–0.90)	0.005	*χ*^2^ = 5.026 (df = 2); *p* = 0.081	17/37	Age	0.89 (0.80–0.95)	0.004	*χ*^2^ = 0.711 (df = 2); *p* = 0.701
		PMI	1.31 (1.02–1.89)	0.072			PMI	1.17 (0.97–1.51)	0.148	
		GluN2A	0.98 (0.95–1.00)	0.087			GluN2A	1.00 (0.98–1.02)	0.827	
		CAMKIIα	1.02 (0.99–1.06)	0.212			CAMKIIα	1.00 (1.00–1.03)	0.528	

Separate models were performed for each defined cluster in the post-mortem dorsolateral prefrontal cortex and hippocampus, respectively. Only the most associated clusters in the post-mortem dorsolateral prefrontal cortex were reported (for the rest of the table please see the Supplementary Table 6). Logistic regression was used to model the probability of having the schizophrenia disease (i.e. outcome), conditioned to the values of the independent variables (i.e. exposures) included into the model as a linear combination. The OR quantifies how many times the risk (i.e. odds) of the disease is higher per one unit increase of each independent variable. OR > 1 means greater odds of association with the exposure and outcome; OR = 1 means there is no association between exposure and outcome and OR < 1 means there is a lower odds of association between the exposure and outcome.

*DLPFC* dorsolateral prefrontal cortex, *HIP* hippocampus, *CTRL* controls, *SCZ* patients with schizophrenia, *OR* odds ratio, *CI* confidence interval, *N.SCZ/total* number of SCZ patients (numerator) and total subjects (denominator) with no missing data for all the variables included in the model (i.e. complete case analysis), *df* degrees of freedom.

*This statistic is based on the model’s residual deviance, which assess the extent to which the likelihood of the “full” model (i.e. which includes age, PMI and pattern-related covariates) exceeds the likelihood of the “reference” model (i.e. which includes age and PMI confounders only). It follows a Chi-square (*χ*^2^) distribution with degrees of freedom equal to the number of parameters in the model (i.e. age, PMI and covariate patterns) minus two (i.e. the number of confounders: age and PMI). When statistically significant (*p* < 0.05), this statistic suggests that the full model, which included both confounders and the covariates of interest outperforms the “reference” model, which included confounders only.

As already shown, subject’s age at deceased and PMI were strongly predictive of the presence of schizophrenia. When both included in a multivariable logistic regression (reference model), increasing age resulted associated with a lower disease probability (OR = 0.88, 95% CI: 0.80–0.94, *p* = 0.003) whereas increasing PMI was associated with a higher disease probability (OR = 1.22, 95% CI: 1.02–1.54, *p* = 0.048). Both covariates discriminated schizophrenia patients from controls with a very high predictive accuracy (AUC = 0.90, 95% CI: 0.80–0.98) and therefore these were necessarily accounted as strongest confounders for the analyses of multivariable logistic models.

Significant associations were found using molecules from the DLPFC only. Indeed, higher levels of both GluN1 and d-Ser levels resulted still associated to higher disease probability (both OR ≥ 1.00) and significantly outperformed the reference model, which included age and PMI only (*χ*^2^ = 8.515, df = 2, *p* = 0.014 from deviance test). Moreover, the linear combination of L-Glu, mGluR2/3, mGluR5 and EAAT2 (*χ*^2^ = 11.065, df = 4, *p* = 0.026 from deviance test), as well as GluA1 and PSD-95 (*χ*^2^ = 7.945, df = 2, *p* = 0.019 from deviance test) significantly outperformed the reference model, evidencing their contribution for the improvement in statistical association.

### Machine Learning analysis finds pathways of molecules of the glutamatergic synapse that are predictive of schizophrenia in the post-mortem DLPFC and hippocampus

**Fig. 4:**
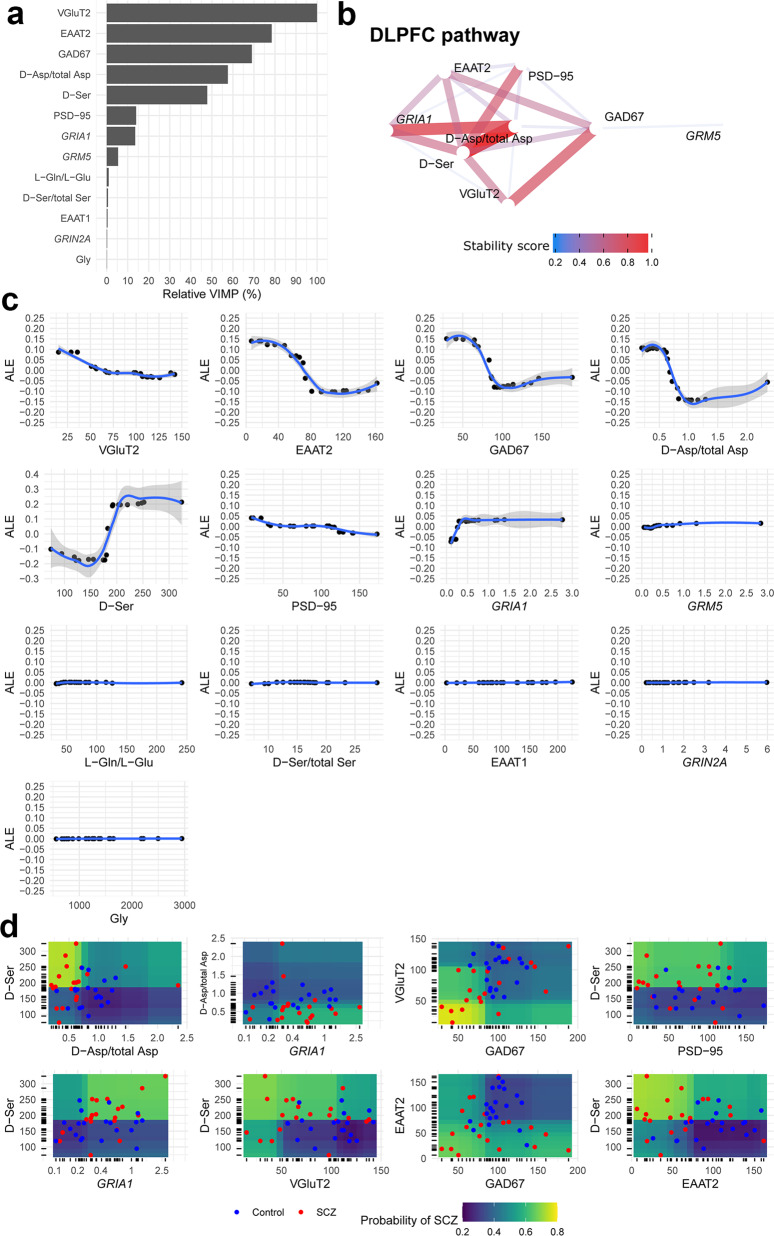
Results from iterative Random Forest detect interactions among glutamatergic synaptic components with predictive signatures of schizophrenia in the DLPFC. **a** Variable importance (VIMP), rescaled from 0% to 100% (relative VIMP) with respect to the maximum achieved value. Only variables (features) with VIMP > 0 are shown and ranked from the most (top) to the less (bottom) important. **b** Pathway of most stable interactions (stability score > 0.20) are reported in network graphs. **c** Accumulated Local Effect (ALE) was computed for each variable with VIMP > 0. ALE describes how the features influence the target (i.e. the predicted probability of having schizophrenia, estimated by iRF) on average. For instance, the ALE estimate of 0.10 at VGLuT2 = 25 means that when the VGluT2 has value 25, then the probability of having the schizophrenia is higher by 0.10 (about 10%) compared to the average probability which is defined at ALE = 0 (i.e. when VGluT2 = 75). The gray band is a confidence band for the regression line fitted in the estimated ALE points. **d** Partial Dependence Plot (PDP) was produced only for those features with top stable interactions (i.e. stability score > 0.60). PDPs show the marginal (total) effect that two features have on the predicted outcome. Colored zones locate those regions at which the disease more likely occurs (high-risk schizophrenia, yellow/green zone) and not occurs (low-risk schizophrenia, blue/violet zone). Individual observations (red: schizophrenia, blue: control) are plotted with respect to each feature combination. mRNA expressions are reported in logarithmic scale.

**Fig. 5:**
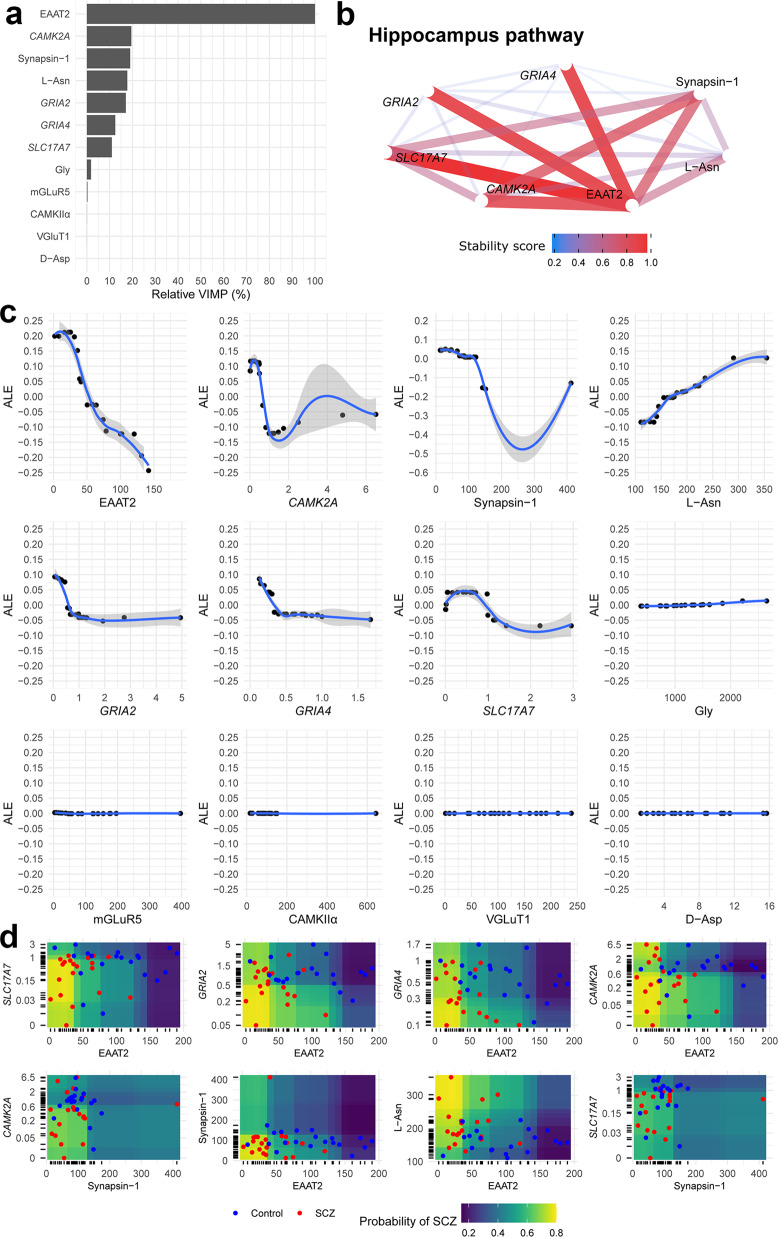
Results from iterative Random Forest detect interactions among glutamatergic synaptic components with predictive signatures of schizophrenia in the hippocampus. **a** Variable importance (VIMP), rescaled from 0% to 100% (relative VIMP) with respect to the maximum achieved value, is reported on panel (**a**). Only variables (features) with VIMP > 0 are shown and were ranked from the most (top) to the less (bottom) important. **b** Pathway of most stable interactions (i.e. with stability score > 0.20) are reported in network graphs. (**c**) Accumulated Local Effect (ALE) was computed for each variable with VIMP > 0. ALE describes how the features influence the target (i.e. the predicted probability of having schizophrenia, estimated by iRF) on average. For instance, the ALE estimate of −0.25 at EAAT2 = 150 means that when the EAAT2 has value 150, then the probability of having the schizophrenia is lower by 0.25 (about 25%) compared to the average probability which is defined at ALE = 0 (i.e. when EAAT2 = 50). The gray band is a confidence band for the regression line fitted in the estimated ALE points. **d** Partial Dependence Plot (PDP) was produced only for those features with top stable interactions (i.e. stability score > 0.60). PDPs show the marginal (total) effect that two features have on the predicted outcome. Colored zones locate those regions at which the disease more likely occurs (high-risk schizophrenia, yellow/green zone) and not occurs (low-risk schizophrenia, blue/violet zone). Individual observations (red: schizophrenia, blue: control) are plotted with respect to each feature combination. mRNA expressions are reported in logarithmic scale.

Results from iterative Random Forests (iRFs) at the last iteration are reported both in the DLPFC (Fig. 4) and hippocampus (Fig. 5). A graphical plot of the Brier Scores achieved by iRFs in the OOB data at different parameter values (i.e. during the “tuning phase”) is reported in Supplementary Fig. 3 whereas results of a finer grid search for the optimal number of iterations and regularization factor, among iRFs with 100,000 trees, is reported in Supplementary Table 7. As for DLPFC, iRF achieved a relatively small prediction error (Brier Score = 0.186) and a very high discriminatory power (AUC = 0.80, 95% CI: 0.65–0.92). The molecules that mostly contributed to discriminate schizophrenia from control were: VGluT2, EAAT2, GAD67, d-Asp/total Asp, d-Ser, PSD-95, *GRIA1* and *GRM5* whereas the ones that barely contributed to the discrimination were: l-Gln/l-Glu ratio, d-Ser/total Ser, EAAT1, *GRIN2A* and Gly (Fig. 4a). The pathway of the most stable interactions (recurrently recovered in the trees of the forest and with a stability score > 0.20) were graphically represented in Fig. 4b. Prevalent interactions were found between d-Ser and d-Asp/total Asp, between d-Asp/total Asp and *GRIA1*, between d-Ser and the following molecules: PSD-95, *GRIA1*, VGluT2, EAAT2, and GAD67. To understand whether the relationship between the levels of each single molecule and the probability of having schizophrenia is linear, monotonic or more complex, accumulated local effect (ALE) was estimated with respect to the most important variables only (Fig. 4c). Higher VGluT2 levels were associated to a linear decreasing in schizophrenia probability whereas a clearly non-linear relationship (sigmoid curves) was found with respect to EAAT2, GAD67, d-Asp/total Asp, and d-Ser. As for EAAT2, GAD67, d-Asp/total Asp, the probability of schizophrenia was higher with respect to their lower levels and then become lower with respect to their higher levels. In contrast, for d-Ser, the probability of schizophrenia was lower with respect to its lower levels and then become higher with respect to its higher levels. Moreover, to investigate in which “direction” the molecules interact with one another (Fig. 4b), i.e. locating those regions at which the disease more likely occurred, partial dependence plots (PDPs) were produced only for those features with top stable interactions (stability score > 0.60). As shown in Fig. 4d, subjects with d-Asp/total Asp ratio lower than 1.00 and with d-Ser levels greater than 200 nmol/g of tissue achieved about 80% probability of having schizophrenia whereas, in the opposite region, such probability was dramatically reduced about to 20%.

As for hippocampus, iRF achieved a smaller prediction error (Brier Score = 0.165) and a higher discriminatory power (AUC = 0.85, 95% CI: 0.71–0.95). The molecules that mostly contributed to discriminate schizophrenia from controls were EAAT2, CAMK2A, Synapsin-1, l-Asn, *GRIA2*, *GRIA4*, and *SLC17A7* whereas the ones that barely contributed to the discrimination were: Gly, mGluR5, CaMKIIα, VGluT1, and d-Asp (Fig. 5a). The pathway of the most stable interactions was represented in Fig. 5b. The most recurrent interactions were found with respect to EAAT2 levels. As shown by ALE plots (Fig. 5c), higher EAAT2 levels (% of control) were associated to a relevant linear decrease in schizophrenia probability, higher l-Asn levels (nmol/g of tissue) were associated to a relevant linear increase in schizophrenia probability whereas a non-linear relationship was detected with respect to the rest of the molecules. Moreover, PDP (Fig. 5d) suggested that subjects with lower levels of EAAT2 in conjunction with lower levels of *SLC17A7* or Synapsin-1 were more likely to achieve higher probability of schizophrenia.

Moreover, Classification And Regression Tree (CART) showed that in the DLPFC, subjects with d-Ser ≥ 185 nmol/g achieved 85% chance of having the schizophrenia whereas those with d-Ser < 185 nmol/g were only 21% more likely to have the disease (Supplementary Fig. 4). In the hippocampus, subjects with CAMK2A expression < 0.79 and (at the same time) with *GRIA2* expression < 0.64 achieved 95% chance of having the schizophrenia whereas those with CAMK2A expression ≥ 0.79 and EAAT2 ≥ 37 % were more likely to do not have the disease at all (probability of 0%). The discriminatory accuracy achieved by both CARTs was AUC = 0.73 (95% CI: 0.60–0.85) and AUC = 0.92 (95% CI: 0.83–0.99) for DLPFC and hippocampus data, respectively.

## Discussion

Despite the recognized role of the glutamate system in the molecular pathophysiology of schizophrenia^5,30^, studies in post-mortem brains analyzing multiple clusters of molecules related to the glutamate signaling are missing. In the present work, we exploited this strategy, measuring both mRNA and protein expression of fundamental molecules at the glutamatergic synapse, as well as neuroactive amino acids levels in the post-mortem DLPFC and hippocampus of schizophrenia patients and non-psychiatric controls.

Paradoxically, univariate analyses revealed that, after the adjustment for multiple comparisons, none of the molecules we investigated was significantly different between schizophrenia patients and controls in the post-mortem DLPFC and hippocampus. Nonetheless, when shifting the approach from a univariate perspective to multivariate hypothesis-driven and hypothesis-free strategies, we discovered that the odds of belonging to the schizophrenia instead of the control group were significantly affected by variations in amino acids and glutamate-related synaptic elements. The hypothesis-driven strategy revealed three molecular patterns including: (1) the GluN1 subunit of the NMDAR and its ligand, d-Ser, the major NMDAR co-agonist in the forebrain^31^; (2) l-Glu, the metabotropic receptors, mGluR2/3, mGluR5, along with the glutamate transporter EAAT2; (3) the scaffolding protein PSD-95 and the AMPAR subunit GluA1. On the other hand, the hypothesis-free strategy evidenced two robust and stable molecule pathways, one in the DLPFC and one in the hippocampus, whose levels were correlated within stable statistical interactions and predictive of each individual classification as a schizophrenia patient or control.

In detail, the cluster including the GluN1 subunit of NMDARs, prevalently distributed at the post-synaptic portion of the synapse, and its respective ligand, D-Ser, is of interest to both the pathophysiology and the treatment of schizophrenia. First of all, a reduction of GluN1 in the post-mortem PFC of patients with schizophrenia has been reported in different cohorts^23,32,33^, and has been suggested to modify NMDAR stoichiometry, therefore being responsible for the endogenous NMDAR deficit reported in schizophrenia^32^. On the other hand, multiple lines of evidence indicate d-Ser as a major modulator of NMDAR function with potential therapeutic effects^31^ and in vivo significant implication as an auditory^34^ and cognitive enhancer^35,36^ in schizophrenia. Moreover, different studies pointed to d-Ser as a potential biomarker^37–39^, given its reduced levels in the serum and CSF of schizophrenia patients, compared to controls^40–42^. However, other investigations and meta-analysis study reported no alterations in d-Ser levels in the blood and CSF^43,44^, as well as in the post-mortem brain of schizophrenia patients^45^, as also found in the present work. Consistent with the relevance of this issue, other investigations call to clarify such controversial results.

By adopting the same approach as above, we also identified a cluster of post-synaptic proteins, namely the ionotropic AMPAR GluA1 subunit and PSD-95, which have been both implicated in schizophrenia^46,47^ and are synergically involved in the post-synaptic glutamate signaling along with synaptic neuroplasticity rearrangements of relevance to schizophrenia pathophysiology^48,49^. Indeed, this cluster of molecules is highly representative of the molecular machinery responsible for the architecture and functional modulation of the post-synaptic density in schizophrenia patients^50^. Specifically, the PSD-95 is an integral part of the post-synaptic density and have attracted interest in schizophrenia pathophysiology based on GWASs^51^. Moreover, PSD-95 is involved in the targeting, clustering, and dynamic retention of AMPARs to post-synaptic densities^52^. Therefore, our results confirmed previous reports that changes in glutamate receptors may not be the only molecular event responsible for glutamate signaling perturbation in schizophrenia^5,53,54^ since also alterations at the post-synaptic level downstream receptor activation may contribute to the emergence of schizophrenia pathophysiology.

Another key cluster of molecules discriminating schizophrenia patients and controls included l-Glu, mGluR2/3, mGluR5, along with EAAT2. Such a cluster captures a critical portion of molecular variation at both pre- and post-synaptic side of the glutamatergic synapse. Indeed, mGluR5 is mainly localized at post-synaptic level, where it is implicated in excitatory events mediating neural plasticity and cognitive processes^55^. Importantly, mGluR5 has been linked to schizophrenia pathophysiology^55,56^ and regarded as a potential novel target for antipsychotic therapy with modulator agents^57,58^. On the other hand, mGluR2 and mGluR3 are found in various combinations of pre-synaptic, post-synaptic and glial localizations^59^. Moreover, *GRM3* gene, encoding mGluR3, has been pinpointed as putative harbor for schizophrenia risk variants by structural and functional GWASs^60,61^ and this association was confirmed by a comprehensive meta-analysis including 11,000 subjects. EAAT2 is expressed predominantly in astroglial cells and is regarded as the main glutamate transporter, responsible for the vast majority of glutamate clearance at the glutamate synapse level^62^. Interestingly, in agreement with our data that include mGluR2/3 and EAAT2 in a cluster discriminating schizophrenia and control subjects, previous studies identified reduced EAAT2 expression in the PFC of subjects with high-risk *GRM3* haplotype associated with schizophrenia^63^, and highlighted multiple EAAT2 interactome-associated biological pathways alteration in the disorder^64^. Finally, in line with the strong tendency to reduction of EAAT2 in both the DLPFC and hippocampus of schizophrenia patients, compared with controls, other studies have previously revealed significant decrease in EAAT2 expression in the post-mortem DLPFC^65^ and parahippocampal regions of schizophrenia subjects^66^.

Interestingly, when we used the Machine Learning hypothesis-free analysis, we identified in both DLPFC and hippocampus stable molecule pathways that discriminate schizophrenia patients from non-psychiatric controls, which could not be conceived using the hypothesis-driven approach. Indeed, the latter only allowed for assessing the association between the weighted linear combination of some molecule levels and the presence of the disease, although excluding the possibility to formulate any a priori assumption about the specific molecular patterns underlying such combination. Although possible associations between VGluT2, EAAT2, GAD67, d-Ser, and PSD-95 levels and the presence of the disease in the DLPFC were originally assessed in the hypothesis-driven approach, strongest interactions between d-Ser with d-Asp/total Asp levels, as well as between GAD67 with VGluT2 levels, along with a marginal effect of *GRIA1* and *GRM5* mRNA expressions, were found using only the hypothesis-free approach. Importantly, the Machine Learning algorithm also provided helpful insights into the molecular signatures of schizophrenia at the hippocampus level. Indeed, in such a brain region, surprising strong and stable pairwise interactions of the EAAT2 levels with *SLC17A7* and *GRIA2* transcripts, Synapsin-1 and CAMK2A were detected. Altogether, these results underline that different pattern of multiple interacting proteins both at glutamate pre-synaptic and post-synaptic level could account for discriminating patients from control subjects.

Our study has some weakness. First, our samples of post-mortem brains from patients’ group had significantly longer PMI compared with healthy individuals. Moreover, patients are on average younger than controls. However, this apparent discrepancy is in line with literature reporting a reduced life expectation in patients with schizophrenia^67–69^, compared with the general population. Based on these differences, we corrected our analyses for the potential confounding effect of PMI and age with the further inclusion of such variables in all statistical models we performed. Furthermore, one possible confounder of our results could be represented by the type and dosage of medication. With this regard, at the time of their *exitus*, the patients we analyzed were undergoing antipsychotic treatments with one or more of first-generation (Fluphenazine and Haloperidol) and second-generation antipsychotics (Risperidone, Olanzapine, Aripiprazole, Ziprasidone, Compazine, and Quetiapine) (Supplementary Table 3). Unfortunately, dosages of these antipsychotics were not available and, because of the fragmentary nature of the information we had with this respect, we could not correct results of our analyses for the effect of such medications. Nonetheless, it is worth noticing how evidence suggests that the overall profile of the antipsychotic treatments assumed by the sample we examined, with the exception of Quetiapine, was broadly characterized by comparable levels of D2 receptor blockade^70–74^.

Finally, the reported observations are related to the analysis of total homogenate samples. Therefore, we cannot discriminate the molecules on the basis of their synaptic localization within cell body, synaptosomal or extracellular fraction.

To our knowledge, this is the first work aiming to identify molecular signatures of schizophrenia by looking at multi levels variation of such a large number of elements implicated in the glutamate synapse. In conclusion, our results indicate that changes in the overall landscape of glutamate synapse more than alteration in single molecules underpin the pathophysiology of schizophrenia. This observation suggests, in turn, that future pharmacological strategies aiming to reduce symptoms of schizophrenia by targeting the glutamate system should be directed towards large interactomes operating within such a synapse, more than targeting one single molecule.

## Methods

### Human tissue collection

DLPFC and hippocampus samples from post-mortem brains of non-psychiatric controls and schizophrenia patients (*n* = 20/brain region/clinical condition) were obtained from The Human Brain and Spinal Fluid Resource Center (Los Angeles Healthcare Center, Los Angeles, CA, USA). All tissue collection and processing were carried out under the regulations and licenses of the Human Tissue Authority and in accordance with the Human Tissue Act of 2004. Clinical diagnosis of schizophrenia was made according to DSMIII-R criteria. Frozen tissues were pulverized in liquid nitrogen and stored at −80 °C for subsequent processing.

### HPLC analysis

Post-mortem brain samples were homogenized in 1:10 (w/v) 0.2 M trichloroacetic acid. The samples were sonicated (3 cycles, 10 s each) and centrifuged at 13,000 × *g* for 20 min. Precipitated protein pellets were stored at −80 °C for protein quantification^75^. Samples were then neutralized with 0.2 M NaOH and subjected to pre-column derivatization with *o*-phthaldialdehyde/N-acetyl-l-cysteine in 50% methanol. Diastereoisomer derivatives were resolved on a Simmetry C8 5-μm reversed-phase column (Waters, 4.6 × 250 mm) in isocratic conditions (0.1 M sodium acetate buffer, pH 6.2, 1% tetrahydrofuran, 1 ml/min flow rate)^76^. A washing step in 0.1 M sodium acetate buffer, 3% tetrahydrofuran and 47% acetonitrile, was performed after every single run. Identification and quantification of d-aspartate (d-Asp), l-aspartate (l-Asp), l-glutamate (l-Glu), l-asparagine (l-Asn), d-serine (d-Ser), l-serine (l-Ser), l-glutamine (l-Gln), and glycine (Gly) were based on retention times (mean ± SEM of min: d-Asp = 4.11 ± 0.015, l-Asp = 4.23 ± 0.005, l-Glu = 10.80 ± 0.018, l-Asn = 17.86 ± 0.038, d-Ser = 19.13 ± 0.015, l-Ser = 21.18 ± 0.07, l-Gln = 29.85 ± 0.072, Gly = 44.81 ± 0.12; Fig. 1b) and peak areas and then compared with those associated with external standards. d-Asp peak specificity was also evaluated by selective degradation catalyzed by a recombinant human d-aspartate oxidase^77,78^. Human d-aspartate oxidase enzyme (12.5 μg) was added to the samples, incubated at 30 °C for 3 h, and subsequently derivatized. Total protein content of homogenates was determined by Bradford assay method, after re-solubilization of the trichloroacetic acid precipitated protein pellets. The detected amino acids concentration was then normalized by the total protein content and expressed as nmol/mg protein. d-amino acid/total amino acid ratio was expressed as percentage (%).

### RNA extraction and quantitative RT-PCR analysis

Total RNA was extracted from post-mortem tissues using RNeasy^®^ mini kit (Qiagen, Hilden, Germany) according to the manufacturer’s instructions (Querques et al., 2015). Total RNA was purified to eliminate potentially contaminating genomic DNA using recombinant DNase (Qiagen, Hilden, Germany). RNA integrity number (RIN) of samples was assessed using Agilent 2100 Bioanalyzer Expert (Santa Clara, California, USA) and Biorad Experion Automated electrophoresis Station (Hercules, CA) prior to cDNA synthesis using Transcriptor First Strand cDNA Synthesis kit (Roche Diagnostics, Mannheim, Germany). A total of 1 μg of total RNA of each sample was reverse transcribed with QuantiTect Reverse Transcription (Qiagen, Hilden, Germany) using oligo-dT and random primers according to the manufacturer’s instructions. Quantitative RT-PCR with Real Time ready catalog Assays (Roche Diagnostics) and LightCycler^®^ 480 Probe Master (Roche Diagnostics) was performed on a Light Cycler 480 Real Time PCR thermocycler with 96-well format (Roche Diagnostics). All measurements from each subject were performed in duplicate. The following protocol was used: 10 s for initial denaturation at 95 °C followed by 40 cycles consisting of 10 s at 94 °C for denaturation, 10 s at 60 °C for annealing, and 6 s for elongation at 72 °C temperature^79^. The primers used for *GRIN1*, *GRIN2A*, *GRIN2B*, *GRIA1*, *GRIA2*, *GRIA3*, *GRM1*, *GRM2*, *GRM3*, *GRM5*, *Homer1*, *DLG4*, *GAD1*, *GAD2*, *SLC1A3*, *SLC1A2*, *SLC1A7*, *SLC1A6*, *CAMK2A*, *SYN1* mRNA amplification are listed in Supplementary Table 1. mRNA expression levels were normalized to the mean of two housekeeping genes: *β-actin* (*ACTB*) and *cyclophilin* (*PPIA*). mRNA expression was calculated using the geometric mean of the two reference genes selected and the relative quantification method (2^−ΔΔCt^).

### Western blotting

Frozen, powdered samples from *post-mortem* DLPFC and hippocampus tissues were sonicated in 1% SDS and boiled for 10 min. Aliquots (2 µl) of the homogenate were used for protein determination using a Bio-Rad Protein Assay kit. Equal amounts of total proteins (30 µg) for each sample were loaded on pre-cast 4-20% gradient gel (BioRad Laboratories). Proteins were separated by SDS–PAGE and transferred to PVDF membranes (GE Healthcare) using Trans Blot Turbo System. Membranes were immunoblotted overnight using the following primary antibodies: GluN1, GluN2A, GluN2B, GluA1, GluA2/3, GluA4, mGluR1, mGluR2/3, mGluR5, Homer1b/c, PSD-95, GAD65, GAD67, EAAT1, EAAT2, VGluT1, VGluT2, Synapsin-1, CaMKIIα, Thr-286-P-CaMKIIα (antibodies specimens are listed Supplementary Table 2. Blots were then incubated with α-rabbit or α-mouse horseradish peroxidase conjugated secondary antibodies. Immunoreactivity was detected by enhanced chemiluminescence (ECL) (GE-Healthcare) and quantified by Quantity One software (Bio-Rad). Optical density values were normalized to GAPDH for variations in loading and transfer. Normalized values were then averaged and used for statistical comparisons. All blots derive from the same experiment and were processed in parallel.

### Statistical methods

Data are reported as medians, along with interquartile range (first-third quartiles—IQR), and as absolute and relative frequency (percentages) for continuous and categorical variables, respectively. The normality assumption was assessed by the Shapiro–Wilk test. For continuous variables with right-skewed distribution, statistical analyses were performed using their log-transformed values. Comparisons of clinical characteristics (age at deceased, gender, PMI, pH) between schizophrenia patients and controls were performed using (two-tailed) two-sample *t*-test or Chi-Square statistic with Yates’s correction for continuous and categorical variables, respectively. Age and PMI—adjusted comparisons between schizophrenia patients and controls were performed by ANCOVA models and *p*-values were also corrected for multiple testing, following the Bonferroni method. For *t*-tests and ANCOVAs, *t*-values and *F*-values along with degrees of freedom were also provided, respectively. Furthermore, to assess whether a linear combination of multiple molecules of the synaptic components was predictive of the presence of schizophrenia, a multivariable logistic model, which included both the molecules as main effects and the strongest confounders (i.e., age, PMI) as covariates, was performed and compared to the one which included confounders only by the deviance test. Results were reported as odds ratio (OR), along with their 95% confidence interval (CI). Unknown patterns of multiple molecules of the synaptic components were detected by the Iterative Random Forest (iRF) algorithm^80^, using a complete dataset with imputed missing values. The imputation was performed by the Multivariate imputation by chained equations (MICE) algorithm^81^ with 10 chains of multiple imputations and 50 iterations per chain, using a random forest of 10 trees per each iteration (see the paragraph: “Handling missing values” in the *Supplemental Statistical Methods* section of the Supplemental Information for further details). The iRF is an ensemble of machine learning (model-agnostic) method for classification and regression that operates by constructing a multitude of decision trees. iRF is a generalization of a Random Forest (RF) and is commonly used to train a feature-weighted ensemble of decision trees to detect stable and high-order interactions^80^. As well as in a RF, each decision tree in the iRF is built on a bootstrap sample from the original dataset. The portion of the bootstrap dataset not used for the building of each tree is called Out Of Bag (OOB) data and is employed to get both an unbiased estimate of the RF prediction error (i.e. the Brier Score) and an estimate of a “variable importance” (VIMP). The predicted individual probability of having the disease is computed as the average of all probabilities over all trees in the forest estimated in OOB data for that individual and the Brier Score is computed as the mean squared difference between such predicted probabilities and the actual outcomes. The Brier Score varies from 0 (i.e. RF is perfectly calibrated) to 1 (i.e. RF is perfectly miscalibrated). To address the between groups imbalances (i.e. adjusting the analysis by subjects’ age and PMIs) in the iRFs, new individual weights were estimated following the Inverse Probability Weighting method^82^ (as a first step) and then such Inverse Probability Weights (IPWs) were supplied to iRFs (as a second step). Because of this, observations with higher IPWs are selected more frequently into each bootstrap sample, which will be used to build each decision tree of the forest, with respect to those with lower IPWs (see the paragraph: “Handling imbalance data between patients and controls in the iRF algorithms” in the *Supplemental Statistical Methods* section of the Supplemental Information for further details). In the iRF algorithm, a RF will be iteratively performed *K* times. At the first iteration, a subsample of candidate variables (i.e. features) will be randomly selected at each split of a decision tree. On the basis of the variable importance and a regularization factor, new weights will be assigned to each variable so that, at the next iteration, variables with higher weights will be selected with higher probability than the others. Therefore, at the last iteration, the iRF will include regularized trees and decision rules extracted from such feature-weighted RF are mapped^83^. This mapping allows to identify prevalent interactions in the RF through a computationally efficient algorithm (i.e. generalized Random Intersection Trees–RIT–algorithm^80^) that searches for high-order interactions in binary data. A bagging step eventually assesses the stability of recovered interactions with respect to the bootstrap perturbation of the data. The proportion of times (out of B bootstrap samples) an interaction appears as an output of the RIT defines a “stability score” (i.e. 0 = totally instable interaction, 1 = totally stable interaction). The following parameters must be set to enable the iRF training, some of them were fixed in advance whereas some others were determined after a “tuning phase”: (1) the number of random forest iterations: from 1 to 10 iterations were evaluated during the tuning phase; (2) the number of the trees included into the random forest (within each iteration): from 50 to 100,000 trees were evaluated during the tuning phase; (3) the choice of the variable regularization factor, where possible fixed values were: 1.0 (no regularization), 0.9 (weak regularization), 0.8 (moderate regularization), <0.8 (strong regularization) and were evaluated during the tuning phase; (4) the number of randomly chosen features that possibly split at in each node of the tree: this parameter was fixed to seven features; (5) the number of outer-layer bootstrap samples: this parameter was fixed to 30; (6) the node splitting criterion: Gini impurity measure; (7) the minimal node size: it was fixed that the final leaves of each tree in the forest must include at least five subjects. The “tuning phase” consists in a grid search of the optimal parameters combination that minimize the Brier Score achieved by iRF in the OOB data (see the paragraph: “Sensitivity of iRFs algorithms to tuning parameters” in the *Supplemental Statistical Methods* section of the Supplemental Information for further details). Accumulated local effects (ALE) and partial dependence plots (PDP) were performed to better quantify changes in disease probabilities at different variable values and detect the direction of the “most stable” interactions found by the RIT, respectively. To define a single classification rule, a tree-growing algorithm that recursively splits data into subgroups (i.e. Classification And Regression Tree) was eventually performed and the choice of tree size was determined on the basis of 10-fold cross validation of the prediction error. The discriminatory ability of both models and machine-learning algorithms was assessed by the Area Under the ROC Curve (AUC), along with its 95% CI computed with 1000 stratified bootstrap replicates. Further details about statistical analysis can be found in Supplemental Information. A *p*-value < 0.05 was considered for statistical significance. Statistical analyses and plots were performed using R foundation for statistical computing, Vienna, Austria (version 4.04).

### Reporting summary

Further information on research design is available in the Nature Research Reporting Summary linked to this article.

## Supplementary information

REPORTING SUMMARY

Supplemental Information

## Data Availability

The data that support the findings of this study are available from the corresponding author upon reasonable request.

## References

[1] Crabtree GW, Gogos JA (2014). Synaptic plasticity, neural circuits, and the emerging role of altered short-term information processing in schizophrenia. Front. Synaptic Neurosci..

[2] Begre S, Koenig T (2008). Cerebral disconnectivity: an early event in schizophrenia. Neuroscientist.

[3] Meyer-Lindenberg A (2010). From maps to mechanisms through neuroimaging of schizophrenia. Nature.

[4] Moreno-De-Luca D, Martin CL (2021). All for one and one for all: heterogeneity of genetic etiologies in neurodevelopmental psychiatric disorders. Curr. Opin. Genet. Dev..

[5] Coyle JT, Ruzicka WB, Balu DT (2020). Fifty years of research on schizophrenia: the ascendance of the glutamatergic synapse. Am. J. Psychiatry.

[6] Baselmans BML, Yengo L, van Rheenen W, Wray NR (2021). Risk in relatives, heritability, snp-based heritability, and genetic correlations in psychiatric disorders: a review. Biol. Psychiatry.

[7] Bayes A (2011). Characterization of the proteome, diseases and evolution of the human postsynaptic density. Nat. Neurosci..

[8] Hall D, Gogos JA, Karayiorgou M (2004). The contribution of three strong candidate schizophrenia susceptibility genes in demographically distinct populations. Genes Brain Behav..

[9] Kirov G (2004). Strong evidence for association between the dystrobrevin binding protein 1 gene (DTNBP1) and schizophrenia in 488 parent-offspring trios from Bulgaria. Biol. Psychiatry.

[10] Network & Pathway Analysis Subgroup of the Psychiatric Genomics, C. Corrigendum: Psychiatric genome-wide association study analyses implicate neuronal, immune and histone pathways. *Nat. Neurosci*. **18**, 1861 (2015). 10.1038/nn1215-1861c 26605885

[11] Huerta I, McCullumsmith RE, Haroutunian V, Gimenez-Amaya JM, Meador-Woodruff JH (2006). Expression of excitatory amino acid transporter interacting protein transcripts in the thalamus in schizophrenia. Synapse.

[12] McCullumsmith RE, Meador-Woodruff JH (2002). Striatal excitatory amino acid transporter transcript expression in schizophrenia, bipolar disorder, and major depressive disorder. Neuropsychopharmacology.

[13] O’Donovan SM, Sullivan CR, McCullumsmith RE (2017). The role of glutamate transporters in the pathophysiology of neuropsychiatric disorders. NPJ Schizophr..

[14] Spangaro M (2018). Neurobiology of cognitive remediation in schizophrenia: Effects of EAAT2 polymorphism. Schizophr. Res..

[15] Chen Q (2018). Schizophrenia polygenic risk score predicts mnemonic hippocampal activity. Brain.

[16] Rampino A (2017). Association of functional genetic variation in PP2A with prefrontal working memory processing. Behav. Brain Res..

[17] Lieberman JA (2018). Hippocampal dysfunction in the pathophysiology of schizophrenia: a selective review and hypothesis for early detection and intervention. Mol. Psychiatry.

[18] Balu DT, Coyle JT (2015). The NMDA receptor ‘glycine modulatory site’ in schizophrenia: d-serine, glycine, and beyond. Curr. Opin. Pharmacol..

[19] Errico F, Nuzzo T, Carella M, Bertolino A, Usiello A (2018). The emerging role of altered d-aspartate metabolism in schizophrenia: new insights from preclinical models and human studies. Front. Psychiatry.

[20] Zalcman G, Federman N, Romano A (2018). CaMKII isoforms in learning and memory: localization and function. Front. Mol. Neurosci..

[21] Mirza FJ, Zahid S (2018). The role of synapsins in neurological disorders. Neurosci. Bull..

[22] Uno Y, Coyle JT (2019). Glutamate hypothesis in schizophrenia. Psychiatry Clin. Neurosci..

[23] Errico F (2013). Decreased levels of d-aspartate and NMDA in the prefrontal cortex and striatum of patients with schizophrenia. J. Psychiatr. Res..

[24] Nuzzo T (2017). Decreased free d-aspartate levels are linked to enhanced d-aspartate oxidase activity in the dorsolateral prefrontal cortex of schizophrenia patients. NPJ Schizophr..

[25] Errico F, Cuomo M, Canu N, Caputo V, Usiello A (2020). New insights on the influence of free d-aspartate metabolism in the mammalian brain during prenatal and postnatal life. Biochim. Biophys. Acta Proteins Proteom..

[26] Mothet JP (2000). D-serine is an endogenous ligand for the glycine site of the N-methyl-d-aspartate receptor. Proc. Natl Acad. Sci. USA.

[27] Herring BE, Silm K, Edwards RH, Nicoll RA (2015). Is aspartate an excitatory neurotransmitter?. J. Neurosci..

[28] Ohi K (2015). Glutamate networks implicate cognitive impairments in schizophrenia: genome-wide association studies of 52 cognitive phenotypes. Schizophr. Bull..

[29] Zhou H (2018). Genome-wide association study identifies glutamate ionotropic receptor GRIA4 as a risk gene for comorbid nicotine dependence and major depression. Transl. Psychiatry.

[30] Balu DT (2016). The NMDA receptor and schizophrenia: from pathophysiology to treatment. Adv. Pharmacol..

[31] Coyle, J. T., Balu, D. & Wolosker, H. d-Serine, the shape-shifting NMDA receptor co-agonist. *Neurochem. Res.*10.1007/s11064-020-03014-1 (2020). 10.1007/s11064-020-03014-1 PMC7313399 32189130

[32] Weickert CS (2013). Molecular evidence of N-methyl-d-aspartate receptor hypofunction in schizophrenia. Mol. Psychiatry.

[33] Beneyto M, Meador-Woodruff JH (2008). Lamina-specific abnormalities of NMDA receptor-associated postsynaptic protein transcripts in the prefrontal cortex in schizophrenia and bipolar disorder. Neuropsychopharmacology.

[34] Koshiyama D (2019). Gamma-band auditory steady-state response is associated with plasma levels of d-serine in schizophrenia: an exploratory study. Schizophr. Res..

[35] Panizzutti R (2019). Association between increased serum d-serine and cognitive gains induced by intensive cognitive training in schizophrenia. Schizophr. Res..

[36] Guercio GD, Panizzutti R (2018). Potential and challenges for the clinical use of d-serine as a cognitive enhancer. Front. Psychiatry.

[37] Javitt DC, Zukin SR, Heresco-Levy U, Umbricht D (2012). Has an angel shown the way? Etiological and therapeutic implications of the PCP/NMDA model of schizophrenia. Schizophr. Bull..

[38] Lin CH, Yang HT, Lane HY (2019). d-glutamate, d-serine, and d-alanine differ in their roles in cognitive decline in patients with Alzheimer’s disease or mild cognitive impairment. Pharmacol. Biochem. Behav..

[39] MacKay, M. -A. B. et al. D-serine: potential therapeutic agent and/or biomarker in schizophrenia and depression? **10**, 10.3389/fpsyt.2019.00025 (2019). 10.3389/fpsyt.2019.00025 PMC6372501 30787885

[40] Bendikov I (2007). A CSF and postmortem brain study of d-serine metabolic parameters in schizophrenia. Schizophr. Res..

[41] Hashimoto K (2005). Reduced d-serine to total serine ratio in the cerebrospinal fluid of drug naive schizophrenic patients. Prog. Neuropsychopharmacol. Biol. Psychiatry.

[42] Hashimoto K (2003). Decreased serum levels of d-serine in patients with schizophrenia: evidence in support of the N-methyl-d-aspartate receptor hypofunction hypothesis of schizophrenia. Arch. Gen. Psychiatry.

[43] Brouwer A, Luykx JJ, van Boxmeer L, Bakker SC, Kahn RS (2013). NMDA-receptor coagonists in serum, plasma, and cerebrospinal fluid of schizophrenia patients: a meta-analysis of case-control studies. Neurosci. Biobehav. Rev..

[44] Fuchs SA (2008). Cerebrospinal fluid d-serine and glycine concentrations are unaltered and unaffected by olanzapine therapy in male schizophrenic patients. Eur. Neuropsychopharmacol..

[45] Kumashiro S, Hashimoto A, Nishikawa T (1995). Free d-serine in post-mortem brains and spinal cords of individuals with and without neuropsychiatric diseases. Brain Res..

[46] Iasevoli F, Tomasetti C, Buonaguro EF, de Bartolomeis A (2014). The glutamatergic aspects of schizophrenia molecular pathophysiology: role of the postsynaptic density, and implications for treatment. Curr. Neuropharmacol..

[47] Coley AA, Gao WJ (2018). PSD95: a synaptic protein implicated in schizophrenia or autism?. Prog. Neuropsychopharmacol. Biol. Psychiatry.

[48] Xu W (2011). PSD-95-like membrane associated guanylate kinases (PSD-MAGUKs) and synaptic plasticity. Curr. Opin. Neurobiol..

[49] Kessels HW, Malinow R (2009). Synaptic AMPA receptor plasticity and behavior. Neuron.

[50] Kaizuka T, Takumi T (2018). Postsynaptic density proteins and their involvement in neurodevelopmental disorders. J. Biochem..

[51] Focking M (2015). Proteomic and genomic evidence implicates the postsynaptic density in schizophrenia. Mol. Psychiatry.

[52] Zeng M (2019). Phase separation-mediated TARP/MAGUK complex condensation and AMPA receptor synaptic transmission. Neuron.

[53] Bondi C, Matthews M, Moghaddam B (2012). Glutamatergic animal models of schizophrenia. Curr. Pharm. Des..

[54] Li CT, Yang KC, Lin WC (2018). Glutamatergic dysfunction and glutamatergic compounds for major psychiatric disorders: evidence from clinical neuroimaging studies. Front. Psychiatry.

[55] Menard C, Quirion R (2012). Successful cognitive aging in rats: a role for mGluR5 glutamate receptors, homer 1 proteins and downstream signaling pathways. PLoS ONE.

[56] Piers TM (2012). Translational concepts of mGluR5 in synaptic diseases of the brain. Front. Pharmacol..

[57] Matosin N, Fernandez-Enright F, Lum JS, Newell KA (2017). Shifting towards a model of mGluR5 dysregulation in schizophrenia: consequences for future schizophrenia treatment. Neuropharmacology.

[58] Matosin N, Frank E, Deng C, Huang XF, Newell KA (2013). Metabotropic glutamate receptor 5 binding and protein expression in schizophrenia and following antipsychotic drug treatment. Schizophr. Res..

[59] Petralia RS, Wang YX, Niedzielski AS, Wenthold RJ (1996). The metabotropic glutamate receptors, mGluR2 and mGluR3, show unique postsynaptic, presynaptic and glial localizations. Neuroscience.

[60] Harrison PJ, Lyon L, Sartorius LJ, Burnet PW, Lane TA (2008). The group II metabotropic glutamate receptor 3 (mGluR3, mGlu3, GRM3): expression, function and involvement in schizophrenia. J. Psychopharmacol..

[61] Maj C, Minelli A, Giacopuzzi E, Sacchetti E, Gennarelli M (2016). The role of metabotropic glutamate receptor genes in schizophrenia. Curr. Neuropharmacol..

[62] Lauriat TL, McInnes LA (2007). EAAT2 regulation and splicing: relevance to psychiatric and neurological disorders. Mol. Psychiatry.

[63] Egan MF (2004). Variation in GRM3 affects cognition, prefrontal glutamate, and risk for schizophrenia. Proc. Natl Acad. Sci. USA.

[64] Asah, S., Alganem, K., McCullumsmith, R. E. & O’Donovan, S. M. A bioinformatic inquiry of the EAAT2 interactome in postmortem and neuropsychiatric datasets. *Schizophr. Res.*10.1016/j.schres.2020.03.018 (2020). 10.1016/j.schres.2020.03.018 PMC7494586 32197935

[65] Ohnuma T, Augood SJ, Arai H, McKenna PJ, Emson PC (1998). Expression of the human excitatory amino acid transporter 2 and metabotropic glutamate receptors 3 and 5 in the prefrontal cortex from normal individuals and patients with schizophrenia. Brain Res. Mol. Brain Res..

[66] Ohnuma T (2000). Gene expression of metabotropic glutamate receptor 5 and excitatory amino acid transporter 2 in the schizophrenic hippocampus. Brain Res. Mol. Brain Res..

[67] Laursen TM (2019). Causes of premature mortality in schizophrenia. Curr. Opin. Psychiatry.

[68] Olfson M, Gerhard T, Huang C, Crystal S, Stroup TS (2015). Premature mortality among adults with schizophrenia in the United States. JAMA Psychiatry.

[69] Wildgust HJ, Hodgson R, Beary M (2010). The paradox of premature mortality in schizophrenia: new research questions. J. Psychopharmacol..

[70] de Boer JN (2020). Language in schizophrenia: relation with diagnosis, symptomatology and white matter tracts. NPJ Schizophr..

[71] Kessler RM (2006). Occupancy of striatal and extrastriatal dopamine D2 receptors by clozapine and quetiapine. Neuropsychopharmacology.

[72] Caravaggio F (2020). What proportion of striatal D2 receptors are occupied by endogenous dopamine at baseline? A meta-analysis with implications for understanding antipsychotic occupancy. Neuropharmacology.

[73] Yilmaz Z (2012). Antipsychotics, dopamine D(2) receptor occupancy and clinical improvement in schizophrenia: a meta-analysis. Schizophr. Res..

[74] de Greef R, Maloney A, Olsson-Gisleskog P, Schoemaker J, Panagides J (2011). Dopamine D2 occupancy as a biomarker for antipsychotics: quantifying the relationship with efficacy and extrapyramidal symptoms. AAPS J..

[75] Nuzzo T (2019). The levels of the NMDA receptor co-agonist d-serine are reduced in the substantia nigra of MPTP-lesioned macaques and in the cerebrospinal fluid of Parkinson’s disease patients. Sci. Rep..

[76] Nuzzo, T. et al. Dysfunctional d-aspartate metabolism in BTBR mouse model of idiopathic autism. *Bba-Proteins Proteom.* **1868**, ARTN 140531 (2020). 10.1016/j.bbapap.2020.140531 32853769

[77] Katane M (2017). Structure function relationships in human d-aspartate oxidase: characterisation of variants corresponding to known single nucleotide polymorphisms. Bba-Proteins Proteom..

[78] Katane M (2018). Rat d-aspartate oxidase is more similar to the human enzyme than the mouse enzyme. Bba-Proteins Proteom..

[79] De Rosa A (2020). Prenatal expression of d-aspartate oxidase causes early cerebral d-aspartate depletion and influences brain morphology and cognitive functions at adulthood. Amino Acids.

[80] Basu S, Kumbier K, Brown JB, Yu B (2018). Iterative random forests to discover predictive and stable high-order interactions. Proc. Natl Acad. Sci. USA.

[81] van Buuren, S. & Groothuis-Oudshoorn, K. Mice: multivariate imputation by chained equations in R. *J. Stat. Softw.* **45**, 1–67 (2011).

[82] Cole SR, Hernan MA (2008). Constructing inverse probability weights for marginal structural models. Am. J. Epidemiol..

[83] Deng, H. T. & Runger, G. Feature selection via regularized trees. In: The 2012 International Joint Conference on Neural Networks (IJCNN), 1–8. 10.1109/IJCNN.2012.6252640 (2012).

