# Supplementary material for: Machine Learning algorithm unveils glutamatergic alterations in the post-mortem schizophrenia brain
Source: Schizophrenia (Heidelb). 2022 Feb 25;8(1):8. doi: 10.1038/s41537-022-00231-1 (PMC8881508; doi:10.1038/s41537-022-00231-1)
Supplement: Supplementary file 1 — REPORTING SUMMARY [file 41537_2022_231_MOESM1_ESM.pdf]

nature portfolio

Corresponding author(s): Alessandro Usiello
NPJSCHZ-00705R

Last updated by author(s): Sep 24, 2021

Reporting Summary

Nature Portfolio wishes to improve the reproducibility of the work that we publish. This form provides structure for consistency and transparency in reporting. For further information on Nature Portfolio policies, see our Editorial Policies and the Editorial Policy Checklist.

Statistics

For all statistical analyses, confirm that the following items are present in the figure legend, table legend, main text, or Methods section.

n/a	Confirmed	
☐	☒	The exact sample size (n) for each experimental group/condition, given as a discrete number and unit of measurement
☐	☒	A statement on whether measurements were taken from distinct samples or whether the same sample was measured repeatedly
☐	☒	The statistical test(s) used AND whether they are one- or two-sided<br>*Only common tests should be described solely by name; describe more complex techniques in the Methods section.*
☐	☒	A description of all covariates tested
☐	☒	A description of any assumptions or corrections, such as tests of normality and adjustment for multiple comparisons
☐	☒	A full description of the statistical parameters including central tendency (e.g. means) or other basic estimates (e.g. regression coefficient) AND variation (e.g. standard deviation) or associated estimates of uncertainty (e.g. confidence intervals)
☐	☒	For null hypothesis testing, the test statistic (e.g. F, t, r) with confidence intervals, effect sizes, degrees of freedom and P value noted<br>*Give P values as exact values whenever suitable.*
☒	☐	For Bayesian analysis, information on the choice of priors and Markov chain Monte Carlo settings
☒	☐	For hierarchical and complex designs, identification of the appropriate level for tests and full reporting of outcomes
☒	☐	Estimates of effect sizes (e.g. Cohen's d, Pearson's r), indicating how they were calculated

Our web collection on statistics for biologists contains articles on many of the points above.

Software and code

Policy information about availability of computer code

Data collection	All software used in this study for data collection are either commercially available or open source.
Data analysis	All software used in this study for data analysis are either commercially available or open source.

For manuscripts utilizing custom algorithms or software that are central to the research but not yet described in published literature, software must be made available to editors and reviewers. We strongly encourage code deposition in a community repository (e.g. GitHub). See the Nature Portfolio guidelines for submitting code & software for further information.

Data

Policy information about availability of data

All manuscripts must include a data availability statement. This statement should provide the following information, where applicable:

- Accession codes, unique identifiers, or web links for publicly available datasets
- A description of any restrictions on data availability
- For clinical datasets or third party data, please ensure that the statement adheres to our policy

All reagents and experimental data are available from the authors upon request.

Field-specific reporting

Please select the one below that is the best fit for your research. If you are not sure, read the appropriate sections before making your selection.

☒ Life sciences ☐ Behavioural & social sciences ☐ Ecological, evolutionary & environmental sciences

For a reference copy of the document with all sections, see nature.com/documents/nr-reporting-summary-flat.pdf

Life sciences study design

All studies must disclose on these points even when the disclosure is negative.

Sample size	Sufficient sample sizes were chosen for each experiment to determine whether the outcome was statistically significant.
Data exclusions	There are data exclusions for technical reasons or exhausted samples.
Replication	All attempts to replicate experiments were successful.
Randomization	Randomly selected samples were allocated into experimental groups.
Blinding	Blinding was not implemented in this study.

Reporting for specific materials, systems and methods

We require information from authors about some types of materials, experimental systems and methods used in many studies. Here, indicate whether each material, system or method listed is relevant to your study. If you are not sure if a list item applies to your research, read the appropriate section before selecting a response.

Materials & experimental systems

n/a	Involved in the study	
☐	☒	Antibodies
☒	☐	Eukaryotic cell lines
☒	☐	Palaeontology and archaeology
☒	☐	Animals and other organisms
☒	☐	Human research participants
☒	☐	Clinical data
☒	☐	Dual use research of concern

Methods

n/a	Involved in the study	
☒	☐	ChIP-seq
☒	☐	Flow cytometry
☒	☐	MRI-based neuroimaging

Antibodies

Antibodies used

GluN1 - Cell Signaling (Cat. Number 5704S)
GluN2A - Sigma-Aldrich (Cat. Number G9038)
GluN2B - Cell Signaling Technology (Cat. Number 4212S)
GluA1 - Phospho-Solution (Cat. Number AB1504)
GluA2/3 - Millipore (Cat. Number 07-598)
GluA4 - Millipore (Cat. Number AB1508)
mGluR1 - Millipore (Cat. Number 12551)
mGluR2/3 - Millipore (Cat. Number 06-676)
mGluR5 - Abcam (Cat. Number AB76316)
Homer 1b/c - Santa Cruz Biotechnology (Cat. Number SC-20807)
PSD-95 BD - Transduction Laboratories (Cat. Number 610496)
GAD65/67 - Millipore (Cat. Number ABN904)
EAAT1 - Abcam (Cat. Number AB181036)
EAAT2 - Abcam (Cat. Number AB41621)
VGluT1 - Cell Signalling (Cat. Number 12331S)
VGluT2 - Millipore (Cat. Number MAB5504)
CAMKIIα - Millipore (Cat. Number 05532)
Thr-268-P-CAMKIIα - Millipore (Cat. Number 05-533)
Synapsin-1 - Novus Biologicals (Cat. Number NB300-104)
GAPDH - Santa Cruz Biotechnology (Cat. Number SC32233)

Validation

GluN1 Cell Signaling (Cat. Number 5704S) - Species Reactivity: Human, Mouse, Rat – Application: Western Blotting and Immunoprecipitation

GluN2A Sigma-Aldrich (Cat. Number G9038) - Species Reactivity: Human, Mouse, Rat – Application: Western Blotting

GluN2B Cell Signaling Technology (Cat. Number 4212S) - Species Reactivity: Human, Mouse, Rat – Application: Western Blotting

GluA1 Phospho-Solution (Cat. Number AB1504) - Species Reactivity: Demonstrated to react with human, mouse, and rat. Predicted to react with equine and platypus based on 100% sequence homology – Application: Western Blotting, Immunoprecipitation and Immunohistochemistry (Paraffin)

GluA2/3 Millipore (Cat. Number 07-598) - Species Reactivity: Human, Monkey, Mouse, Rat, Chicken – Application: Western Blotting and Immunoprecipitation

GluA4 Millipore (Cat. Number AB1508) - Species Reactivity: The immunogen sequence shares 100% conservation with chicken, mouse, rat, oppossum, human, chimp, and monkey– Application: Western Blotting, Immunoprecipitation and Immunohistochemistry (Paraffin), Immunocytochemistry

mGluR1 Millipore (Cat. Number 12551) - Species Reactivity: Human, Mouse, Rat – Application: Western Blotting, Immunohistochemistry (Paraffin), Immunofluorescence (Frozen) and Immunoprecipitation

mGluR2/3 Millipore (Cat. Number 06-676) - Species Reactivity: Mouse, Rat – Application: Western Blotting, Immunohistochemistry and Immunoprecipitation

mGluR5 Abcam (Cat. Number AB76316) - Species Reactivity: Human, Mouse, Rat (Notes: The Human species recommendation is based on the WB results. We do not guarantee IHC for Human species) – Application: Western Blotting, Immunohistochemistry and Electron Microscopy

Homer 1b/c Santa Cruz Biotechnology (Cat. Number SC-20807) - Species Reactivity: Bovine, Human, Mouse, Rat, Pig – Application: Western Blotting, Immunohistochemistry

PSD-95 BD Transduction Laboratories (Cat. Number 610496) - Species Reactivity: Mouse, Rat – Application: Western Blotting, Immunohistochemistry and Immunoprecipitation

GAD65/67 Millipore (Cat. Number ABN904) - Species Reactivity: Human, Mouse, Rat – Application: Western Blotting and Immunohistochemistry (Paraffin)

EAAT1 Abcam (Cat. Number AB181036) - Species Reactivity: Human, Mouse, Rat – Application: Western Blotting, Immunohistochemistry and Immunocytochemistry

EAAT2 Abcam (Cat. Number AB41621) - Species Reactivity: Mouse, Rat – Application: Western Blotting and Immunocytochemistry

VGluT1 Cell Signalling (Cat. Number 12331S) - Species Reactivity: Mouse, Rat (Note: Species predicted to react based on 100% sequence homology Human) – Application: Western Blotting

VGluT2 Millipore (Cat. Number MAB5504) - Species Reactivity: Human, Mouse, Rat - Application: Western Blotting, Immunohistochemistry

CAMKIIα Millipore (Cat. Number 05532) - Species Reactivity: Bovine, Rat - Application: Immunohistochemistry, Western Blotting

Thr-268-P-CAMKIIα Millipore (Cat. Number 05-533) - Species Reactivity: Rat - Application: Western Blotting

Synapsin-1 Novus Biologicals (Cat. Number NB300-104) - Species Reactivity: Human, Mouse, Rat, Guinea Pig, Primate - Application: Western Blotting, Immunocytochemistry/Immunofluorescence, Immunohistochemistry, Immunohistochemistry-Frozen, Immunohistochemistry-Paraffin, Immunoprecipitation, Immunohistochemistry Free-Floating

GAPDH Santa Cruz Biotechnology (Cat. Number SC32233) - Species Reactivity: Mouse, Rat, Human, Rabbit and Xenopus laevis - Application: Western Blotting, Immunoprecipitation and Immunofluorescence
