# Supplementary material for: Machine Learning algorithm unveils glutamatergic alterations in the post-mortem schizophrenia brain
Source: Schizophrenia (Heidelb). 2022 Feb 25;8(1):8. doi: 10.1038/s41537-022-00231-1 (PMC8881508; doi:10.1038/s41537-022-00231-1)
Supplement: Supplementary file 2 — Supplemental Information [file 41537_2022_231_MOESM2_ESM.docx]

**Supplemental Information** for

**Machine Learning algorithm unveils glutamatergic alterations in the *post-mortem* schizophrenia brain**

Arianna De Rosa^1,2,#^, Andrea Fontana^3#,^, Tommaso Nuzzo^1,2,§^, Martina Garofalo^1,2,§^, Anna Di Maio^1, §^, Daniela Punzo^1,$^, Massimiliano Copetti^3^, Alessandro Bertolino^4,5^, Francesco Errico^1,6^, Antonio Rampino^4^, Andrea de Bartolomeis^7^, Alessandro Usiello^1,2,^*

^1^CEINGE Biotecnologie Avanzate, 80145, Naples, Italy; ^2^Dipartimento di Scienze e Tecnologie Ambientali Biologiche e Farmaceutiche, Università degli Studi della Campania "Luigi Vanvitelli”, 81100, Caserta, Italy; ^3^Unit of Biostatistics, Fondazione IRCCS "Casa Sollievo della Sofferenza", 71013, San Giovanni Rotondo, Italy; ^4^Group of Psychiatric Neuroscience, Department of Basic Medical Sciences, Neuroscience and Sense Organs, University of Bari Aldo Moro, 70124, Bari, Italy; ^5^Azienda Ospedaliero-Universitaria Consorziale Policlinico, 70124, Bari, Italy; ^6^Department of Agricultural Sciences, University of Naples “Federico II”, 80055, Portici, Italy; ^7^Section of Psychiatry Laboratory of Molecular and Translational Psychiatry, Department of Neuroscience, Reproductive Science and Odontostomatology School of Medicine, University "Federico II", 80131, Naples, Italy.

^#^These authors share the first authorship.

^§^These authors contributed equally to this work.

^$^Present address: Center for Epigenetics and Metabolism, U1233 INSERM, Department of Biological Chemistry, University of California, Irvine, Irvine, CA 92697, USA.

*Corresponding author:

Alessandro Usiello, PhD: Department of Environmental, Biological and Pharmaceutical Sciences and Technologies, University of Campania “Luigi Vanvitelli”, Via A. Vivaldi, 43, 81100 Caserta, Italy, and CEINGE Biotecnologie Avanzate, Naples, Italy; Phone: +39 0813737879, email: [usiello@ceinge.unina.it](mailto:usiello@ceinge.unina.it).

**Running title:** *Glutamatergic synapse in schizophrenia*

**Supplementary Methods**

*Supplemental statistical methods*

The scope of this section is to provide further statistical details about (1) how the data was preprocessed and Inverse Probability Weights are estimated before being supplied to the Iterative Random Forest (iRF) algorithms; (2) how does the Random Forest (RF) algorithm work and the extension to iRF (a rapid overview); 3) the sensitivity of iRFs algorithms to tuning parameters.

(1) Data preprocessing

Before running the iRF algorithms, the following issues must be addressed:

- How to handle missing values in the original dataset

- How to appropriately account for age and post-mortem interval (PMI) imbalance between schizophrenia patients (SCZ) and controls (CTRL) in the iRF algorithms

1a – Handling missing values

The original dataset includes missing values for some molecules (i.e. proteins, mRNA and amino acids) required as an input for all statistical models (i.e. ANCOVA and logistic regression) and iRF algorithms. As for the 52 candidate variables collected in the dorsolateral prefrontal cortex (DLPFC), 30 of them (57.7%) had complete data (i.e. no missing values) whereas for the other 22 variables, the percentage of missing values ranged from 2.5% to 12.5% (median percentage of missing values: 2.5%), depending on the considered variable. As for the 52 candidate variables collected in the hippocampus (HIP), 26 of them (50.0%) had complete data whereas for the other 26 variables, the percentage of missing values ranged from 2.5% to 25.5% (median percentage of missing values: 2.5%), depending on the considered variable. As missing values were merely produced because of technically unsatisfactory samples, the missingness is not related to the values of other variables. Therefore, it is assumed that such data is “Missing Completely At Random”. As for statistical models, because each model usually includes a rather small number of variables (chosen a priori), the listwise deletion strategy (complete-case analysis) can be safely employed. However, for iRF algorithms this was not the case because the whole vector of 52 candidate variables must be passed as input variables. Indeed, if a complete-case analysis was performed a very important data loss would be observed: only 17 and 14 of 40 observations would be available for the analysis using DLPFC and HIP data, respectively. To this reason, iRF algorithms were performed on two “augmented” separate datasets with imputed missing values: one including variables collected in DLPFC and the other one including those collected in HIP. Multivariate Imputation by Chained Equations algorithm was used to impute (in a single shot) missing values in the two datasets. Specifically, 10 chains of multiple imputations were created with 50 iterations per chain using a random forest of 10 trees per each iteration. Convergence of the model was assessed by Rhat statistics ^1^. For each of the two original datasets, 10 datasets with imputed values were therefore created (10 imputed datasets for DLPFC and 10 imputed datasets HIP). To get a single imputed dataset, the median and the mode of the distribution of the 10 imputed values was computed for continuous and categorical variables, respectively.

1b – Handling imbalance data between patients and controls in the iRF algorithms

There are several possibilities for handling imbalances between groups of patients with respect to some covariates of interest. The more classic approach is to put the confounders as covariates into each model directly, so that the estimated effect would be "adjusted for" the presence of the confounders. However, this approach is suitable only for generalized linear models (such as ANCOVA and logistic regression). Another approach is based on units reweighting. Indeed, the following fundamental implicit assumption is made every time a data analysis is performed: all subjects (i.e. statistical units) have the same importance, all of them counts in the same way. That is, the subject "A" counts one, the subject "B" counts one in the dataset, and so on. In other words, subjects “A” and “B” have the same importance and therefore the collected data on both subjects also has the same weight. Now what if this were not the case? Returning to SCZ and controls groups, it is clear that the SCZ group tends to be characterized by younger subjects with a higher PMI. So providing a "greater weight" to the older SCZ patients with lower PMI equals to assign "greater copies" of elderly subjects with low PMI in the SCZ group and, if so, this group would be perfectly balanced in terms of age and PMI. On the contrary, in the group of controls (characterized by older people and with a lower PMI), providing a "greater weight" to the younger subjects with higher PMI, controls group would be perfectly balanced in terms of age and PMI. To define new individual weights, we used the Inverse Probability Weighting (IPW) method. Briefly, the individual probability of having SCZ (“Prob (SCZ)”) is estimated for each subject using a logistic model that includes Age and PMI covariates. In subjects that actually have SCZ this probability remains unchanged. However, in subjects that actually do not have SCZ (i.e. controls), the complementary probability is considered, that is, of not having the disease, and therefore defined as 1-Prob (SCZ). Once estimated the individual probabilities, the new individual weights are estimated by taking the inverse of such probabilities: the new weights in patients with SCZ are 1 / prob (SCZ) whereas the new weights in controls are 1 / (1 - prob (SCZ)). The initial and final (new) weights are reported, for each subject, in the following table:

| **ID** | **Diagnosis**  **group** | **Age** | **PMI** | **Initial weights** | **New weights (IPW)*** |
| --- | --- | --- | --- | --- | --- |
| 1 | CTRL | 47 | 12.5 | 1.000 | 5.724466 |
| 2 | CTRL | 66 | 17.3 | 1.000 | 2.136439 |
| 3 | CTRL | 92 | 23.3 | 1.000 | 1.144362 |
| 4 | CTRL | 84 | 11.8 | 1.000 | 1.039606 |
| 5 | CTRL | 70 | 11.8 | 1.000 | 1.229364 |
| 6 | CTRL | 87 | 9.3 | 1.000 | 1.016499 |
| 7 | CTRL | 58 | 9.0 | 1.000 | 1.590798 |
| 8 | CTRL | 68 | 10.5 | 1.000 | 1.227366 |
| 9 | CTRL | 80 | 14.0 | 1.000 | 1.101516 |
| 10 | CTRL | 76 | 16.0 | 1.000 | 1.250006 |
| 11 | CTRL | 75 | 11.5 | 1.000 | 1.115369 |
| 12 | CTRL | 66 | 13.3 | 1.000 | 1.511179 |
| 13 | CTRL | 64 | 17.5 | 1.000 | 2.520060 |
| 14 | CTRL | 80 | 12.0 | 1.000 | 1.068084 |
| 15 | CTRL | 83 | 17.6 | 1.000 | 1.143008 |
| 16 | CTRL | 79 | 14.0 | 1.000 | 1.115084 |
| 17 | CTRL | 61 | 19.5 | 1.000 | 4.302145 |
| 18 | CTRL | 70 | 12.0 | 1.000 | 1.238712 |
| 19 | CTRL | 72 | 12.2 | 1.000 | 1.193312 |
| 20 | CTRL | 81 | 14.5 | 1.000 | 1.098951 |
| 21 | SCZ | 46 | 21.7 | 1.000 | 1.029725 |
| 22 | SCZ | 55 | 10.7 | 1.000 | 1.827272 |
| 23 | SCZ | 53 | 20.5 | 1.000 | 1.090907 |
| 24 | SCZ | 70 | 24.0 | 1.000 | 1.381245 |
| 25 | SCZ | 35 | 35.7 | 1.000 | 1.000456 |
| 26 | SCZ | 32 | 12.3 | 1.000 | 1.033555 |
| 27 | SCZ | 61 | 28.0 | 1.000 | 1.055448 |
| 28 | SCZ | 41 | 20.8 | 1.000 | 1.019001 |
| 29 | SCZ | 50 | 13.7 | 1.000 | 1.242663 |
| 30 | SCZ | 18 | 26.3 | 1.000 | 1.000354 |
| 31 | SCZ | 46 | 11.6 | 1.000 | 1.223477 |
| 32 | SCZ | 29 | 27.3 | 1.000 | 1.001151 |
| 33 | SCZ | 60 | 10.3 | 1.000 | 2.677877 |
| 34 | SCZ | 75 | 14.9 | 1.000 | 5.395252 |
| 35 | SCZ | 77 | 14.7 | 1.000 | 6.878929 |
| 36 | SCZ | 77 | 26.5 | 1.000 | 1.556835 |
| 37 | SCZ | 24 | 12.8 | 1.000 | 1.011131 |
| 38 | SCZ | 62 | 12.2 | 1.000 | 2.475413 |
| 39 | SCZ | 52 | 15.6 | 1.000 | 1.213382 |
| 40 | SCZ | 55 | 12.6 | 1.000 | 1.566025 |

ID: Identifier (progressive) number for each recruited subject; CTRL: Control subjects; SCZ: patients with schizophrenia; *inverse probabilities estimated from a multivariable logistic regression model

The vector of the estimated IPW was supplied as a separate parameter in the iRF algorithm, so that individuals with larger weights will be selected with higher probability in the bootstrap samples during the trees building.

(2) How does the Random Forest (RF) algorithm work

A Random Forest (RF) is a popular machine learning algorithm which consists in an ensemble of decision trees. In RFs, there is no need for cross-validation or a separate test set to get an unbiased estimate of the test set error: it is estimated internally during the run because each tree is built on a bootstrap sample (where observations are randomly extracted with replacement) from the original dataset. About one-third of the observations are left out of the bootstrap sample and not used in the construction of each tree: this is called Out Of Bag (OOB) data. Because IPWs are supplied by the user, observations with higher IPWs are selected more frequently into each bootstrap sample with respect to those with lower IPWs. The tree-growing algorithm recursively splits the bootstrap sample data into subgroups, choosing the best binary split for each considered variable at issue, to identify the most homogeneous sets within each tree node and the most heterogeneous ones between the nodes (Gini Impurity measure). To split each tree node, if there are M candidate input variables in the dataset, only m<<M variables are randomly selected (every time, out of the M) to be considered for that split. The tree is grown until the reaching of the minimal node size (stop criteria defined by the user). OOB data of each tree is used to get a running unbiased estimate of the RF prediction error (i.e. the Brier Score) as trees are added to the forest: for each observation in the OOB data, each tree gives the probability of having the disease. OOB data is also used to get an estimate the variable importance (VIMP): it is defined as the average across all trees of the difference in prediction error before and after the permutation of each variable values in the OOB data within each tree.

The iRF algorithm is a generalization of the RF which will be iteratively performed K times (see *Statistical Methods* section in the main manuscript for further details).

3) Sensitivity of iRFs algorithms to tuning parameters

As already stated in the *Statistical Methods* section in the main manuscript, iRF parameters must be set to enable the algorithm training: some of them were fixed in advance whereas some others were determined after a “tuning phase”. In total, there are 10 parameters (6 are required and 4 are optional) that must be set by the user, as shown in the following table:

|  | **Parameters (description)** | **Setting values** |
| --- | --- | --- |
| **Required** | The number of random forest iterations | Evaluated: from 1 to 10 iterations.  Final choice: 10 for DLPFC data and 7 for HIP data |
|  | The number of the trees included into the forest (within each iteration) | Evaluated: from 50 to 100’000 trees.  Final choice: 100’000 trees |
|  | The number of (randomly chosen) features that possibly split at in each node of the tree | 7 of 52 candidate features (i.e. the square root) were randomly chosen for each tree (default option) |
|  | The number of outer-layer bootstrap samples | 30 |
|  | The splitting criterion | Each node in the tree was split by the feature that minimize the Gini impurity measure |
|  | Minimal node size | The final leaves of each tree in the forest must include at least 5 subjects (about 12.5% of the total sample) |
| **Optional** | Variable regularization factor: regularization works by penalizing new features by multiplying the splitting criterion by a factor, in order to perform an efficient feature selection, so that "regularized trees" are built. The key idea is to penalize selecting a new feature for splitting when its gain (e.g. information gain) is similar to the features used in previous splits | Evaluated: 0.8, 0.9, 1  Final choice: 0.8 |
|  | Weights for sampling of observations. Observations with larger weights will be selected with higher probability in the bootstrap (or subsampled) samples for the trees | The supplied vector of estimated IPW |
|  | Should the forest return a binary classification or an individual probability? In the probability forest, each tree of the forest returns a probability estimate (of having the SCZ) and these estimates are averaged for the forest probability estimate | Probability forests were grown along with the estimation of Brier Score (i.e. prediction error) |
|  | Variable Importance measure | Permutation variable importance (i.e. permuting OOB cases) |

The “tuning phase” consists in a grid search of the optimal parameters combination that minimize the Brier Score achieved by iRF in the OOB data. The parameters whose values were established after the tuning phase were: the number of iRF iterations, the number of trees in the forest and the value of the regularization factor.

As shown in Supplementary Figure 3, the Brier Score: 1) was drastically reduced just passing from the first to the second iteration, independently by the number of trees or regularization factors and achieved lower values in a iRF with at a regularization factor of 0.8; 2) lower volatility is detected at higher number of trees included into the forest. Because of this, the largest number of trees (i.e. 100’000) was finally considered in iRFs both for DLPFC and HIP data. Moreover, among iRFs with 100’000 trees, a finer grid search for the optimal number of iRF iterations and regularization factor was performed (see Supplementary Table 7) and the optimal fitting was achieved for iRFs with 10 and 7 iterations and regularization factor at 0.8 for DLPFC and HIP data, respectively.

**Supplementary figures and tables**

**Supplementary Table 1.** Sybr green primer sequences used for qPCR analysis.

| **Gene** | **Forward primer (5'-3')** | **Reverse primer (5'-3')** |
| --- | --- | --- |
| *GRIN1* | TGCAAGGAGGAGTTCACAGT | AGGTGAAGTTCATGGTCCGT |
| *GRIN2A* | GGAACGTTCTGAAGTGGTGG | GGCAGAAACAATGAGCAGCA |
| *GRIN2B* | TGAAGAGCAGGAGGATGACC | TTTGATGTAACCCGGCTCCT |
| *GRIA1* | GGAAGGACGGGACCAGACAA | AACGATGCGACCAGACAGGG |
| *GRIA2* | TGTGGAGCCAAGGACTCTG | CCCCCGACAAGGATGTAGA |
| *GRIA3* | GCAGAGAAAACCATGTGATACG | GCCAGGTTAACAGCATTTCC |
| *GRIA4* | CCAACTGAATTTTACTTACCCTGAA | GACATGAAAAAGGGCACTAA |
| *GRM1* | CTGGCATGAAGGAGTGCTGAAC | AGCAGCTCACTTCTCCTTTCCG |
| *GRM2* | CGCTCCACTCCGATTCTCT | TGTTGTTCCAAGGGTCCAG |
| *GRM3* | CATGTACACCACGTGCATCA | CATGGTTGTCGTCTGCACTC |
| *GRM5* | ATGACGGTGAGAGGTCTGCTGA | TGATGCCACCAACAGCTTCTCG |
| *Homer1* | GAAAGAACACCTGATGTGACACAG | CCTTTGAGGGTAGCCAGTTCAG |
| *DLG4* | TCCACTCTGACAGTGAGACCGA | CGTCACTGTCTCGTAGCTCAGA |
| *GAD1* | CTGCTCCAGTCTCCAAAGCC | CCGTGAACTTCTGAGCCACT |
| *GAD2* | CGAGGACTCTGGACAGTAGAGG | GATCTTGAGCCCCAGTTTTCTG |
| *SLC1A3* | TTGAACTGAACTTCGGACAAATTA | ATTCCAGCTGCCCCAATACT |
| *SLC1A2* | GTTTCAGCCGCTCGACTC | AACTCACAGGCAGGCACAC |
| *SLC17A7* | GCAAGTACATCGAGGACGCCAT | GCCACGATGATGGCATAGACTG |
| *SLC17A6* | AATCACTCGGCCAGATCTACA | CGTCAGCTCGATTGTCTCC |
| *CAMK2A* | GAGCCATTCTCACCACGATGCT | TGGTGTTGGTGCTCTCTGAGGA |
| *SYN1* | CGATGCCAAATATGACGTGCGTG | AGCATCGCAGAGCCAGTATTGG |
| *ACTB* | TCCTCCCTGGAGAAGAGCTA | CGTGGATGCCACAGGACT |
| *PP1A* | TTCATCTGCACTGCCAAGAC | CACTTTGCCAAACACCACAT |

**Supplementary Table 2.** Primary antibodies used for Western blotting.

| **Protein name** | **MW (kDa)** | **Dilution** | **Manufacturer** |
| --- | --- | --- | --- |
| GluN1 | 120 | 1:1000 | Cell Signaling Technology |
| GluN2A | 180 | 1:1000 | Sigma-Aldrich |
| GluN2B | 190 | 1:500 | Cell Signaling Technology |
| GluA1 | 110 | 1:500 | Phospho-Solution |
| GluA2/3 | 100 | 1:500 | Millipore |
| GluA4 | 100 | 1:1000 | Millipore |
| mGluR1 | 140 | 1:1000 | Millipore |
| mGluR2/3 | 100 | 1:1000 | Millipore |
| mGluR5 | 140/150 | 1:5000 | Abcam |
| Homer1b/c | 45 | 1:1000 | Santa Cruz Biotechnology |
| PSD-95 | 95 | 1:1000 | BD Transduction Laboratories |
| GAD65/67 | 65 | 1:1000 | Millipore |
| EAAT1 | 59 | 1:1000 | Abcam |
| EAAT2 | 62 | 1:10000 | Abcam |
| VGluT1 | 62 | 1:1000 | Cell Signaling |
| VGluT2 | 56 | 1:5000 | Millipore |
| CAMKIIα | 50 | 1:1000 | Millipore |
| Thr-286-P-CAMKIIα | 50 | 1:1000 | Millipore |
| Synapsin-1 | 77 | 1:1000 | Novus Biologicals |
| GAPDH | 37 | 1:1000 | Santa Cruz Biotechnology |

**Supplementary Table 3.** Demographic characteristics, comorbidities, clinical diagnosis of each control subject and schizophrenia patient

| **Control** | | | | | | | **Schizophrenia** | | | | | | | |
| --- | --- | --- | --- | --- | --- | --- | --- | --- | --- | --- | --- | --- | --- | --- |
| *ID* | *Sex* | *Age*  *(years)* | *PMI*  *(hours)* | *pH* | *RIN* | *Clinical Diagnosis* | *ID* | *Sex* | *Age*  *(years)* | *PMI*  *(hours)* | *pH* | *RIN* | *Clinical Diagnosis* | *Antipsychotic medication* |
| *1* | M | 47 | 12.5 | 6.53 | *7.8* | CA (esophagus) with metastases to the liver | *21* | M | 46 | 21.7 | 6.45 | *6.7* | Schizophrenia, Depression, Bipolar, Seizure disorder, Epilepsy, Substance abuse | Quietapine |
| *2* | M | 66 | 17.3 | 6.49 | *5.7* | CA (lung), COPD | *22* | M | 55 | 10.7 | N.A. | *5.4* | Schizophrenia, Suicide, Overdose, Depression, Anxiety, Hypochondriasis | Risperidone, Fluphenazine |
| *3* | F | 92 | 23.3 | 6.75 | *4.6* | CA (uterus, stomach), Congestive hearth failure, Hypertension, Macular degeneration, | *23* | M | 53 | 20.5 | N.A. | *6.1* | Schizophrenia, Depression, Bipolar, CA (lung) Paranoia, Psychosis, Hypertension, Anxiety | Olanzapine, Fluphenazine |
| *4* | M | 84 | 11.8 | 6.79 | *6.7* | CA (stomach), Renal failure, acute, COPD | *24* | M | 70 | 24.0 | 6.56 | *6.9* | Schizophrenia, Paranoia, Aggressive behavior, Dementia, Impulse disorder, Tuberculosis | N.A. |
| *5* | M | 70 | 11.8 | 6.62 | *5.9* | Coronary hearth disease, Leukemia, Type I diabetes, Myocardial infarction, Congestive | *25* | M | 35 | 35.7 | 6.51 | *7.5* | Schizophrenia, Alcohol abuse | Risperidone, Haloperidol |
| *6* | M | 87 | 9.3 | 6.76 | *7.1* | Congestive hearth failure, Atherosclerosis, COPD | *26* | F | 32 | 12.3 | 6.51 | *7.3* | Schizophrenia, Alcohol abuse history | N.A. |
| *7* | M | 58 | 9.0 | 6.32 | *7.2* | CA (colon) | *27* | M | 61 | 28.0 | 6.73 | *6.9* | Schizophrenia, Aggressive behavior, Suicide, Attempts, Anxiety, Tobacco abuse, Asthma | Thioridazine, Fluphenazine, Quietapine, Paliperidone |
| *8* | M | 68 | 10.5 | N.A. | *6.2* | CA (lung), Alcohol abuse, Type I diabetes, Transient Ischemic Attack | *28* | F | 41 | 20.8 | 6.39 | *4.9* | Schizophrenia, Suicide, Stabbing, Psychosis, Disassociated Disorders, Electroconvulsive | Quietapine, Haloperidol, Risperidone |
| *9* | M | 80 | 14.0 | 6.49 | *5* | CA (bladder), Hypertension, Diabetes type II, CVA | *29* | F | 50 | 13.7 | N.A. | *6.9* | Schizophrenia, Suicide, Overdose, Aggressive behavior, Depression, Migraine, Hallucination | Risperidone |
| *10* | M | 76 | 16.0 | 6.55 | *6.4* | CA (lung). Pulmonary emphysema | *30* | M | 18 | 26.3 | 6.72 | *5.9* | Schizophrenia, Mentally retarded (clinical only), Attention Deficit Disorder, Sleep Apnea | Risperidone |
| *11* | M | 75 | 11.5 | 6.6 | *5.2* | CA (prostate) Coronary hearth disease, Hypertension | *31* | M | 46 | 11.6 | 6.41 | *6.5* | Schizophrenia, Suicide, hanging, Alcohol abuse, Substance abuse (not Alcohol), Depression | Quietapine, Risperidone |
| *12* | M | 66 | 13.3 | N.A. | *5.5* | CA (larynx) Metastasis to bone and liver, Type I diabetes | *32* | F | 29 | 27.3 | 6.4 | *6.8* | Schizophrenia, Depression, Seizure Disorder, Attention Deficit Disorder, Aggressive behavior | Aripiprazole, Risperidone |
| *13* | M | 64 | 17.5 | 6.63 | *5.5* | Lymphoma, Coronary Artery Disease | *33* | M | 60 | 10.3 | N.A. | *6.5* | Schizophrenia, Depression, Bipolar, Alcohol abuse, Psychotic disorder, Inappropriate sexual | Ziprasidone, Risperidone |
| *14* | M | 80 | 12.0 | N.A. | *5.7* | CA (kidney) Hypertension, Atrial fibrillation, Macular degeneration, COPD | *34* | F | 75 | 14.9 | N.A. | *3.4* | Schizophrenia, CA (pancreas), Dementia, Therapeutic lobotomy, Dysphagia | Risperidone |
| *15* | F | 83 | 17.6 | 6.41 | *5.2* | CA (breast, uterus, colon), Macular degeneration, Chronic urinary tract infection | *35* | F | 77 | 14.7 | 6.38 | *7* | Schizophrenia, Alcohol abuse, Depression, Bipolar and Seizure disorder | Fluphenazine, Risperidone |
| *16* | F | 79 | 14.0 | N.A. | *6.8* | Coronary hearth disease, Hypertension | *36* | M | 77 | 26.5 | 6.56 | *6.4* | Schizophrenia, Hypertension, Dementia, COPD | Quietapine |
| *17* | M | 61 | 19.5 | 6.29 | *7.4* | Normal | *37* | M | 24 | 12.8 | 6.47 | *7.3* | Schizophrenia, Depression, Substance abuse (not Alcohol), Suicide Attempts, Alcohol abuse | Aripiprazole, Quietapine |
| *18* | M | 70 | 12.0 | N.A. | *5.6* | Renal failure, acute, Diabetes type I | *38* | F | 62 | 12.2 | 6.67 | *1.4* | Schizophrenia, Stroke/CVA, Depression, Hypertension, Diabetes type I | Quietapine |
| *19* | M | 72 | 12.2 | 6.54 | *7.8* | COPD, Pulmonary emphysema | *39* | F | 52 | 15.6 | 6.49 | *7.6* | Schizophrenia, Depression, CA (pancreas), Diabetes Type II, Hypothyroidism, Hypertension | Compazine, Risperidone |
| *20* | F | 81 | 14.5 | N.A. | *7.9* | COPD, Pneumonia, Osteoporosis, Tuberculosis | *40* | M | 55 | 12.6 | N.A. | *8.1* | Schizophrenia, Paranoid Schizophrenia, Infection Bacterial, Psychotic disorder, Hallucination | Fluphenazine, Chlorpromazine |

**Abbreviations:** ID: Identifier (progressive) number for each recruited subject; PMI: post-mortem interval, RIN: RNA Integrity Number; M: male; F: female; CA: carcinoma; COPD: chronic obstructive pulmonary disease; CVA: cerebrovascular accident; N.A.: Not Available.

**Supplementary Table 4.** Comparisons of mRNA expressions (expressed as relative quantification) between patients with schizophrenia and control subjects in the post-mortem dorsolateral prefrontal cortex and hippocampus, respectively. Results are reported as age and *post-mortem* interval adjusted means along with their 95% confidence interval (CI).

|  | **DLPFC** | | | | | | **HIP** | | | | | |
| --- | --- | --- | --- | --- | --- | --- | --- | --- | --- | --- | --- | --- |
| **mRNA** | **CTRL** ^§^ | | **SCZ** ^§^ | | **Statistics** | | **CTRL** ^§^ | | **SCZ** ^§^ | | **Statistics** | |
|  | **Mean**  **(95% CI)** | **Nr** | **Mean**  **(95% CI)** | **Nr** | **F(df_1_,df_2_);**  ***p*-value raw*** | ***p*-value**  **adjusted^#^** | **Mean**  **(95% CI)** | **Nr** | **Mean**  **(95% CI)** | **Nr** | **F(df_1_,df_2_);**  ***p*-value raw*** | ***p*-value**  **adjusted^#^** |
| *GRIN1* | 1.0 (0.6-1.6) | 20 | 1.0 (0.6-1.6) | 20 | F(1,36)=0.008; p=0.928 | 1.000 | 1.1 (0.7-1.8) | 20 | 0.7 (0.4-1.2) | 19 | F(1,35)=1.195; p=0.282 | 1.000 |
| *GRIN2A* | 1.0 (0.7-1.5) | 20 | 1.3 (0.9-1.9) | 20 | F(1,36)=0.801; p=0.377 | 1.000 | 1.1 (0.7-1.7) | 20 | 1.0 (0.7-1.5) | 19 | F(1,35)=0.097; p=0.758 | 1.000 |
| *GRIN2B* | 1.0 (0.6-1.5) | 20 | 1.1 (0.7-1.7) | 20 | F(1,36)=0.147; p=0.704 | 1.000 | 1.2 (0.7-2.0) | 20 | 0.7 (0.4-1.1) | 19 | F(1,35)=2.090; p=0.157 | 1.000 |
| *GRIA1* | 0.3 (0.2-0.5) | 20 | 0.5 (0.3-0.8) | 20 | F(1,36)=1.940; p=0.172 | 1.000 | 0.6 (0.3-1.1) | 20 | 0.3 (0.2-0.6) | 20 | F(1,36)=1.742; p=0.195 | 1.000 |
| *GRIA2* | 1.0 (0.6-1.5) | 20 | 0.9 (0.6-1.4) | 19 | F(1,35)=0.123; p=0.728 | 1.000 | 1.1 (0.7-1.8) | 20 | 0.5 (0.3-0.7) | 20 | F(1,36)=6.226; p=0.017 | 0.364 |
| *GRIA3* | 0.5 (0.3-0.8) | 20 | 0.6 (0.4-1.0) | 20 | F(1,36)=0.631; p=0.432 | 1.000 | 0.6 (0.4-1.1) | 19 | 0.6 (0.4-1.1) | 20 | F(1,35)=0.005; p=0.942 | 1.000 |
| *GRIA4* | 0.6 (0.4-0.8) | 19 | 0.6 (0.4-0.8) | 20 | F(1,35)=0.004; p=0.952 | 1.000 | 0.6 (0.4-0.8) | 20 | 0.3 (0.2-0.5) | 20 | F(1,36)=3.921; p=0.055 | 1.000 |
| *GRM1* | 0.4 (0.3-0.5) | 19 | 0.5 (0.4-0.7) | 20 | F(1,35)=1.556; p=0.221 | 1.000 | 0.4 (0.2-0.7) | 20 | 0.4 (0.2-0.7) | 20 | F(1,36)=0.016; p=0.901 | 1.000 |
| *GRM2* | 0.2 (0.1-0.3) | 19 | 0.2 (0.2-0.4) | 20 | F(1,35)=0.042; p=0.839 | 1.000 | 1.0 (0.6-1.8) | 19 | 0.7 (0.4-1.2) | 20 | F(1,35)=0.966; p=0.332 | 1.000 |
| *GRM3* | 1.2 (0.9-1.6) | 20 | 0.8 (0.6-1.1) | 20 | F(1,36)=2.854; p=0.100 | 1.000 | 0.8 (0.6-1.1) | 20 | 0.6 (0.4-0.7) | 20 | F(1,36)=3.945; p=0.055 | 1.000 |
| *GRM5* | 0.2 (0.2-0.4) | 19 | 0.3 (0.2-0.5) | 20 | F(1,35)=0.777; p=0.384 | 1.000 | 0.7 (0.5-1.1) | 20 | 0.4 (0.2-0.6) | 20 | F(1,36)=3.867; p=0.057 | 1.000 |
| *Homer1* | 1.1 (0.9-1.4) | 20 | 0.8 (0.7-1.1) | 20 | F(1,36)=2.433; p=0.128 | 1.000 | 1.3 (0.9-1.8) | 20 | 0.7 (0.5-0.9) | 20 | F(1,36)=6.787; p=0.013 | 0.279 |
| *DLG4* | 1.2 (0.8-1.8) | 20 | 0.7 (0.5-1.1) | 20 | F(1,36)=2.039; p=0.162 | 1.000 | 1.0 (0.6-1.6) | 19 | 0.7 (0.4-1.2) | 20 | F(1,35)=0.675; p=0.417 | 1.000 |
| *GAD1* | 1.2 (0.7-2.0) | 20 | 0.6 (0.3-1.0) | 19 | F(1,35)=2.473; p=0.125 | 1.000 | 0.9 (0.5-1.3) | 18 | 1.0 (0.7-1.6) | 20 | F(1,34)=0.298; p=0.588 | 1.000 |
| *GAD2* | 1.1 (0.7-1.7) | 20 | 0.9 (0.5-1.4) | 19 | F(1,35)=0.334; p=0.567 | 1.000 | 1.1 (0.7-1.8) | 19 | 0.9 (0.6-1.5) | 20 | F(1,35)=0.148; p=0.703 | 1.000 |
| *SLC1A3* | 1.7 (1.1-2.8) | 20 | 1.1 (0.7-1.8) | 18 | F(1,34)=1.322; p=0.258 | 1.000 | 0.9 (0.6-1.3) | 20 | 0.6 (0.4-0.9) | 20 | F(1,36)=1.501; p=0.229 | 1.000 |
| *SLC1A2* | 0.5 (0.3-0.8) | 19 | 0.5 (0.3-0.8) | 18 | F(1,33)=0.044; p=0.835 | 1.000 | 0.7 (0.4-1.2) | 20 | 0.6 (0.3-0.9) | 20 | F(1,36)=0.300; p=0.587 | 1.000 |
| *SLC17A7* | 1.2 (0.9-1.7) | 20 | 1.3 (0.9-1.8) | 20 | F(1,36)=0.137; p=0.714 | 1.000 | 0.5 (0.2-1.2) | 19 | 0.3 (0.1-0.6) | 20 | F(1,35)=0.718; p=0.402 | 1.000 |
| *SLC17A6* | 0.2 (0.1-0.3) | 18 | 0.2 (0.1-0.3) | 18 | F(1,32)=0.075; p=0.786 | 1.000 | 0.4 (0.2-0.9) | 20 | 0.4 (0.2-0.8) | 19 | F(1,35)=0.102; p=0.751 | 1.000 |
| *CAMK2A* | 0.8 (0.4-1.7) | 20 | 0.8 (0.4-1.7) | 20 | F(1,36)=0.000; p=0.987 | 1.000 | 0.9 (0.4-1.9) | 20 | 0.3 (0.1-0.7) | 20 | F(1,36)=2.798; p=0.103 | 1.000 |
| *SYN1* | 0.4 (0.3-0.7) | 19 | 0.6 (0.4-0.9) | 19 | F(1,34)=0.467; p=0.499 | 1.000 | 0.4 (0.2-0.8) | 20 | 0.5 (0.2-1.2) | 20 | F(1,36)=0.304; p=0.585 | 1.000 |

**Abbreviations:** DLPFC: Dorsolateral prefrontal cortex; HIP: Hippocampus; CTRL: Control subjects; SCZ: patients with schizophrenia; Nr: Number of subjects with non-missing values for each considered variable;

*to test the difference of adjusted means between the two groups, p-values (raw) were computed from ANCOVA models which include the presence of SCZ as the main grouping variable and age and post-mortem interval as confounders; ^#^adjusted p-values correspond to the raw p-values corrected for multiple testing following the Bonferroni method; ^§^mRNA expressions were log-transformed before statistical analysis (i.e. performing ANCOVA models) but adjusted means (and their 95% confidence interval) were exponentiated for ease of interpretation. F(df_1_,df_2_) is the quantile of the F-distribution with df1 and df2 degrees of freedom corresponding to main grouping variable effect.

**Supplementary Table 5** Comparisons of protein levels (expressed as % of control) between patients with schizophrenia and control subjects in the post-mortem dorsolateral prefrontal cortex and hippocampus, respectively. Results are reported as age and *post-mortem* interval adjusted means along with their 95% confidence interval (CI).

|  | **DLPFC** | | | | | | **HIP** | | | | | |
| --- | --- | --- | --- | --- | --- | --- | --- | --- | --- | --- | --- | --- |
| **Protein** | **CTRL** | | **SCZ** | | **Statistics** | | **CTRL** | | **SCZ** | | **Statistics** | |
|  | **Mean (95% CI)** | **Nr** | **Mean (95% CI)** | **Nr** | **F(df_1_,df_2_);**  ***p*-value raw*** | ***p*-value**  **adjusted^#^** | **Mean (95% CI)** | **Nr** | **Mean (95% CI)** | **Nr** | **F(df_1_,df_2_);**  ***p*-value raw*** | ***p*-value**  **adjusted^#^** |
| GluN1 | 110.6 (77.7-143.6) | 20 | 127.2 (94.2-160.2) | 20 | F(1,36)=0.409; p=0.526 | 1.000 | 110.8 (74.6-147.1) | 20 | 119.0 (82.8-155.3) | 20 | F(1,36)=0.083; p=0.775 | 1.000 |
| GluN2A | 117.3 (96.2-138.4) | 20 | 76.0 (54.2-97.8) | 19 | F(1,35)=5.965; p=0.020 | 0.396 | 105.6 (74.4-136.8) | 20 | 78.6 (44.0-113.3) | 17 | F(1,33)=1.070; p=0.309 | 1.000 |
| GluN2B | 106.5 (79.2-133.8) | 20 | 74.6 (47.3-101.9) | 20 | F(1,36)=2.215; p=0.145 | 1.000 | 95.5 (59.2-131.8) | 19 | 108.8 (72.4-145.1) | 19 | F(1,34)=0.215; p=0.646 | 1.000 |
| GluA1 | 104.0 (77.8-130.2) | 20 | 82.4 (56.2-108.6) | 20 | F(1,36)=1.105; p=0.300 | 1.000 | 101.2 (67.3-135.1) | 20 | 90.8 (56.9-124.7) | 20 | F(1,36)=0.155; p=0.696 | 1.000 |
| GluA2/3 | 105.8 (91.7-120.0) | 20 | 88.2 (74.1-102.4) | 20 | F(1,36)=2.519; p=0.121 | 1.000 | 100.0 (77.7-122.2) | 20 | 72.4 (50.1-94.6) | 20 | F(1,36)=2.501; p=0.123 | 1.000 |
| GluA4 | 118.5 (91.4-145.6) | 19 | 78.7 (51.6-105.8) | 19 | F(1,34)=3.504; p=0.070 | 1.000 | 103.2 (73.7-132.7) | 16 | 104.9 (75.4-134.4) | 16 | F(1,28)=0.006; p=0.941 | 1.000 |
| mGluR1 | 102.4 (72.4-132.4) | 19 | 103.6 (72.5-134.6) | 18 | F(1,33)=0.003; p=0.960 | 1.000 | 88.1 (49.0-127.3) | 14 | 158.4 (119.3-197.5) | 14 | F(1,24)=5.435; p=0.028 | 0.569 |
| mGluR2/3 | 108.6 (75.4-141.8) | 20 | 94.9 (60.6-129.2) | 19 | F(1,35)=0.266; p=0.610 | 1.000 | 104.3 (69.2-139.5) | 20 | 85.8 (50.7-121.0) | 20 | F(1,36)=0.448; p=0.508 | 1.000 |
| mGluR5 | 113.0 (81.3-144.8) | 20 | 65.3 (33.5-97.0) | 20 | F(1,36)=3.679; p=0.063 | 1.000 | 108.3 (65.1-151.4) | 20 | 78.5 (35.4-121.7) | 20 | F(1,36)=0.770; p=0.386 | 1.000 |
| Homer 1b/c | 104.8 (90.7-118.9) | 20 | 93.8 (79.7-107.9) | 20 | F(1,36)=0.994; p=0.326 | 1.000 | 105.1 (82.1-128.0) | 20 | 77.4 (54.4-100.3) | 20 | F(1,36)=2.370; p=0.132 | 1.000 |
| PSD-95 | 107.1 (83.3-131.0) | 18 | 72.3 (47.5-97.0) | 17 | F(1,31)=3.295; p=0.079 | 1.000 | 95.7 (59.7-131.7) | 17 | 101.9 (66.0-137.9) | 17 | F(1,30)=0.048; p=0.827 | 1.000 |
| GAD65 | 100.9 (89.4-112.5) | 20 | 83.4 (71.5-95.3) | 19 | F(1,35)=3.693; p=0.063 | 1.000 | 105.5 (85.8-125.2) | 20 | 81.8 (62.1-101.5) | 20 | F(1,36)=2.342; p=0.135 | 1.000 |
| GAD67 | 107.6 (91.0-124.1) | 20 | 82.3 (65.8-98.9) | 20 | F(1,36)=3.774; p=0.060 | 1.000 | 103.0 (81.0-124.9) | 20 | 96.8 (74.8-118.7) | 20 | F(1,36)=0.129; p=0.721 | 1.000 |
| EAAT1 | 87.8 (59.2-116.3) | 19 | 140.4 (109.7-171.0) | 17 | F(1,32)=5.123; p=0.031 | 0.611 | 99.9 (74.1-125.7) | 17 | 116.9 (93.7-140.2) | 20 | F(1,33)=0.769; p=0.387 | 1.000 |
| EAAT2 | 97.0 (76.4-117.5) | 20 | 62.6 (42.0-83.2) | 20 | F(1,36)=4.524; p=0.040 | 0.807 | 97.1 (73.3-120.8) | 20 | 46.4 (21.8-70.9) | 19 | F(1,35)=7.078; p=0.012 | 0.234 |
| VGluT1 | 92.0 (53.8-130.2) | 19 | 142.7 (103.1-182.2) | 18 | F(1,33)=2.734; p=0.108 | 1.000 | 103.4 (69.4-137.5) | 20 | 115.7 (81.7-149.7) | 20 | F(1,36)=0.212; p=0.648 | 1.000 |
| VGluT2 | 99.7 (82.8-116.6) | 20 | 74.4 (57.6-91.3) | 20 | F(1,36)=3.628; p=0.065 | 1.000 | 97.4 (64.2-130.6) | 20 | 68.1 (35.0-101.3) | 20 | F(1,36)=1.263; p=0.268 | 1.000 |
| CAMKIIα | 96.5 (77.6-115.4) | 20 | 101.2 (81.7-120.8) | 19 | F(1,35)=0.096; p=0.758 | 1.000 | 90.0 (37.8-142.2) | 20 | 113.0 (60.8-165.2) | 20 | F(1,36)=0.316; p=0.578 | 1.000 |
| Thr-286-P- CAMKIIα | 104.6 (77.9-131.3) | 20 | 94.6 (67.0-122.3) | 19 | F(1,35)=0.213; p=0.647 | 1.000 | 98.6 (76.9-120.2) | 20 | 88.2 (64.2-112.1) | 17 | F(1,33)=0.342; p=0.562 | 1.000 |
| Synapsin-1 | 101.5 (78.9-124.1) | 20 | 75.4 (52.0-98.7) | 19 | F(1,35)=2.044; p=0.162 | 1.000 | 90.4 (55.8-124.9) | 20 | 101.1 (66.6-135.7) | 20 | F(1,36)=0.157; p=0.694 | 1.000 |

**Abbreviations:** DLPFC: Dorsolateral prefrontal cortex; HIP: Hippocampus; CTRL: Control subjects; SCZ: patients with schizophrenia; Nr: Number of subjects with non-missing values for each considered variable;

*to test the difference of adjusted means between the two groups, p-values (raw) were computed from ANCOVA models which include the presence of SCZ as the main grouping variable and age and post-mortem interval as confounders; ^#^adjusted p-values correspond to the raw p-values corrected for multiple testing following the Bonferroni method. F(df_1_,df_2_) is the quantile of the F-distribution with df1 and df2 degrees of freedom corresponding to main grouping variable effect.

**Supplementary Table 6.** This is the completion of the **Table 3**, reported in the main manuscript. Results from multivariable logistic regression models which include a linear combination of multiple molecules of synaptic components (chosen a priori) as main covariates along with age and *post-mortem* interval (PMI) as confounders. Separate models were performed for each defined cluster in the post-mortem dorsolateral prefrontal cortex and hippocampus, respectively.

|  | **DLPFC** | | | | | **HIP** | | | | |
| --- | --- | --- | --- | --- | --- | --- | --- | --- | --- | --- |
| **Cluster** | **N.SCZ/total** | **Variable** | **OR (95%CI)** | ***p*-value** | **Deviance test**  **statistic;**  ***p*-value^*^** | **N.SCZ/total** | **Variable** | **OR (95%CI)** | ***p*-value** | **Deviance test**  **statistic;**  ***p*-value^*^** |
| mGluR1 + mGluR5 + Homer1 b/c | 18/37 | Age | 0.86 (0.77-0.94) | 0.003 | χ^2^=1.776 (df=3); p=0.620 | 14/28 | Age | 0.83 (0.66-0.94) | 0.026 | χ^2^=6.844 (df=3); p=0.077 |
|  |  | PMI | 1.26 (1.00-1.75) | 0.091 |  |  | PMI | 1.30 (0.93-2.35) | 0.248 |  |
|  |  | mGluR1 | 1.00 (0.98-1.02) | 0.922 |  |  | mGluR1 | 1.03 (1.01-1.06) | 0.054 |  |
|  |  | mGluR5 | 0.99 (0.97-1.01) | 0.233 |  |  | mGluR5 | 0.99 (0.98-1.01) | 0.508 |  |
|  |  | Homer 1b/c | 1.00 (0.95-1.04) | 0.905 |  |  | Homer 1b/c | 1.01 (0.97-1.06) | 0.572 |  |
| PSD-95 +  Thr-286-P-CAMKIIα + CAMKIIα | 16/34 | Age | 0.80 (0.63-0.91) | 0.010 | χ^2^=1.783 (df=3); p=0.619 | 14/31 | Age | 0.87 (0.74-0.96) | 0.021 | χ^2^=3.514 (df=3); p=0.319 |
|  |  | PMI | 1.42 (1.10-2.14) | 0.028 |  |  | PMI | 1.46 (1.10-2.48) | 0.046 |  |
|  |  | PSD-95 | 0.97 (0.92-1.01) | 0.232 |  |  | PSD-95 | 1.00 (0.97-1.02) | 0.749 |  |
|  |  | Thr-286-P-CAMKIIα | 1.01 (0.97-1.05) | 0.625 |  |  | Thr-286-P-CAMKIIα | 0.97 (0.91-1.00) | 0.160 |  |
|  |  | CAMKIIα | 1.02 (0.95-1.09) | 0.498 |  |  | CAMKIIα | 0.97 (0.91-1.02) | 0.220 |  |
| GluA1 + Homer 1b/c | 20/40 | Age | 0.87 (0.76-0.94) | 0.006 | χ^2^=1.518 (df=2); p=0.468 | 20/40 | Age | 0.88 (0.79-0.95) | 0.005 | χ^2^=0.556 (df=2); p=0.757 |
|  |  | PMI | 1.16 (0.93-1.54) | 0.249 |  |  | PMI | 1.19 (0.98-1.51) | 0.107 |  |
|  |  | GluA1 | 0.99 (0.95-1.01) | 0.379 |  |  | GluA1 | 1.00 (0.98-1.01) | 0.679 |  |
|  |  | Homer 1b/c | 1.00 (0.95-1.06) | 0.933 |  |  | Homer 1b/c | 0.99 (0.97-1.02) | 0.565 |  |
| GluN2B + CAMKIIα | 19/39 | Age | 0.86 (0.75-0.93) | 0.004 | χ^2^=1.232 (df=2); p=0.540 | 19/38 | Age | 0.84 (0.71-0.92) | 0.006 | χ^2^=3.647 (df=2); p=0.161 |
|  |  | PMI | 1.24 (1.02-1.61) | 0.053 |  |  | PMI | 1.25 (1.00-1.68) | 0.080 |  |
|  |  | GluN2B | 0.99 (0.96-1.01) | 0.344 |  |  | GluN2B | 1.02 (1.00-1.04) | 0.091 |  |
|  |  | CAMKIIα | 1.01 (0.98-1.04) | 0.473 |  |  | CAMKIIα | 1.00 (1.00-1.03) | 0.414 |  |
| GluA1 + CAMKIIα | 19/39 | Age | 0.83 (0.69-0.91) | 0.004 | χ^2^=2.897 (df=2); p=0.235 | 20/40 | Age | 0.88 (0.79-0.94) | 0.003 | χ^2^=0.381 (df=2); p=0.826 |
|  |  | PMI | 1.19 (0.96-1.57) | 0.147 |  |  | PMI | 1.21 (1.00-1.53) | 0.075 |  |
|  |  | GluA1 | 0.98 (0.95-1.00) | 0.127 |  |  | GluA1 | 1.00 (0.98-1.01) | 0.632 |  |
|  |  | CAMKIIα | 1.02 (0.98-1.06) | 0.336 |  |  | CAMKIIα | 1.00 (0.99-1.02) | 0.703 |  |
| mGluR5 + CAMKIIα | 19/39 | Age | 0.85 (0.73-0.92) | 0.003 | χ^2^=2.418 (df=2); p=0.298 | 20/40 | Age | 0.88 (0.80-0.95) | 0.003 | χ^2^=0.578 (df=2); p=0.749 |
|  |  | PMI | 1.23 (1.02-1.60) | 0.060 |  |  | PMI | 1.23 (1.02-1.55) | 0.045 |  |
|  |  | mGluR5 | 0.99 (0.97-1.00) | 0.183 |  |  | mGluR5 | 1.00 (0.99-1.01) | 0.499 |  |
|  |  | CAMKIIα | 1.01 (0.98-1.05) | 0.385 |  |  | CAMKIIα | 1.00 (0.99-1.02) | 0.721 |  |
| L-glutamate + VGluT1 + VGluT2 | 18/37 | Age | 0.87 (0.77-0.95) | 0.010 | χ^2^=4.216 (df=3); p=0.239 | 19/39 | Age | 0.89 (0.80-0.95) | 0.006 | χ^2^=1.642 (df=3); p=0.650 |
|  |  | PMI | 1.20 (0.93-1.69) | 0.198 |  |  | PMI | 1.24 (1.00-1.64) | 0.082 |  |
|  |  | L-glutamate | 1.00 (1.00-1.00) | 0.286 |  |  | L-glutamate | 1.00 (1.00-1.00) | 0.408 |  |
|  |  | VGluT1 | 1.01 (1.00-1.04) | 0.178 |  |  | VGluT1 | 1.00 (0.99-1.02) | 0.785 |  |
|  |  | VGluT2 | 0.98 (0.94-1.01) | 0.243 |  |  | VGluT2 | 0.99 (0.97-1.01) | 0.332 |  |
| GAD65 + GAD67 +  L-glutamate + L-glutamine + L-glutamine/L-glutamate ratio | 19/39 | Age | 0.87 (0.74-0.95) | 0.014 | χ^2^=6.237 (df=5); p=0.284 | 19/39 | Age | 0.82 (0.66-0.92) | 0.008 | χ^2^=7.762 (df=5); p=0.170 |
|  |  | PMI | 1.12 (0.88-1.49) | 0.391 |  |  | PMI | 1.13 (0.86-1.63) | 0.447 |  |
|  |  | GAD65 | 0.97 (0.89-1.05) | 0.485 |  |  | GAD65 | 0.94 (0.86-1.00) | 0.102 |  |
|  |  | GAD67 | 0.99 (0.93-1.05) | 0.752 |  |  | GAD67 | 0.96 (0.91-1.00) | 0.066 |  |
|  |  | L-glutamate | 1.00 (1.00-1.00) | 0.156 |  |  | L-glutamate | 1.00 (1.00-1.01) | 0.103 |  |
|  |  | L-glutamine | 1.00 (1.00-1.00) | 0.217 |  |  | L-glutamine | 1.00 (0.99-1.00) | 0.156 |  |
|  |  | L-Gln/L-Glu ratio | 1.09 (0.97-1.26) | 0.166 |  |  | L-Gln/L-Glu ratio | 1.08 (0.97-1.29) | 0.298 |  |
| D-aspartate + GluN2A + GluN2B | 19/39 | Age | 0.81 (0.65-0.92) | 0.011 | χ^2^=6.254 (df=3); p=0.100 | 14/29 | Age | 0.81 (0.65-0.92) | 0.015 | χ^2^=6.305 (df=3); p=0.098 |
|  |  | PMI | 1.25 (0.99-1.76) | 0.098 |  |  | PMI | 1.36 (0.97-2.19) | 0.121 |  |
|  |  | D-aspartate | 0.93 (0.74-1.15) | 0.529 |  |  | D-aspartate | 0.74 (0.47-0.99) | 0.090 |  |
|  |  | GluN2A | 0.96 (0.91-0.99) | 0.058 |  |  | GluN2A | 0.99 (0.95-1.03) | 0.558 |  |
|  |  | GluN2B | 1.02 (0.99-1.06) | 0.214 |  |  | GluN2B | 1.02 (1.00-1.06) | 0.171 |  |

**Abbreviations:** DLPFC: Dorsolateral prefrontal cortex; HIP: Hippocampus; CTRL: Controls; SCZ: patients with schizophrenia; OR: Odds Ratio; CI: Confidence Interval; N.SCZ/total: number of SCZ patients (numerator) and total subjects (denominator) with no missing data for all the variables included in the model (i.e. complete case analysis); df: degrees of freedom.

Logistic regression was used to model the probability of having the schizophrenia disease (i.e. outcome), conditioned to the values of the independent variables (i.e. exposures) included into the model as a linear combination. The OR quantifies how many times the risk (i.e. odds) of the disease is higher per one unit increase of each independent variable. OR > 1 means greater odds of association with the exposure and outcome; OR = 1 means there is no association between exposure and outcome and OR < 1 means there is a lower odds of association between the exposure and outcome.

*This statistic is based on the model’s residual deviance, which assess the extent to which the likelihood of the “full” model (i.e. which includes age, PMI and pattern-related covariates) exceeds the likelihood of the “reference” model (i.e. which includes age and PMI confounders only). It follows a Chi-square distribution (χ^2^) with degrees of freedom equal to the number of parameters in the model (i.e. age, PMI and covariate patterns) minus two (i.e. the number of confounders: age and PMI). When statistically significant (p<0.05), this statistic suggests that the full model, which included both confounders and the covariates of interest outperforms the “reference” model, which included confounders only.

**Supplementary Figure 1.** Raw blots of figure 3a. Abbreviations: CTRL = Control; SCZ = schizophrenia.


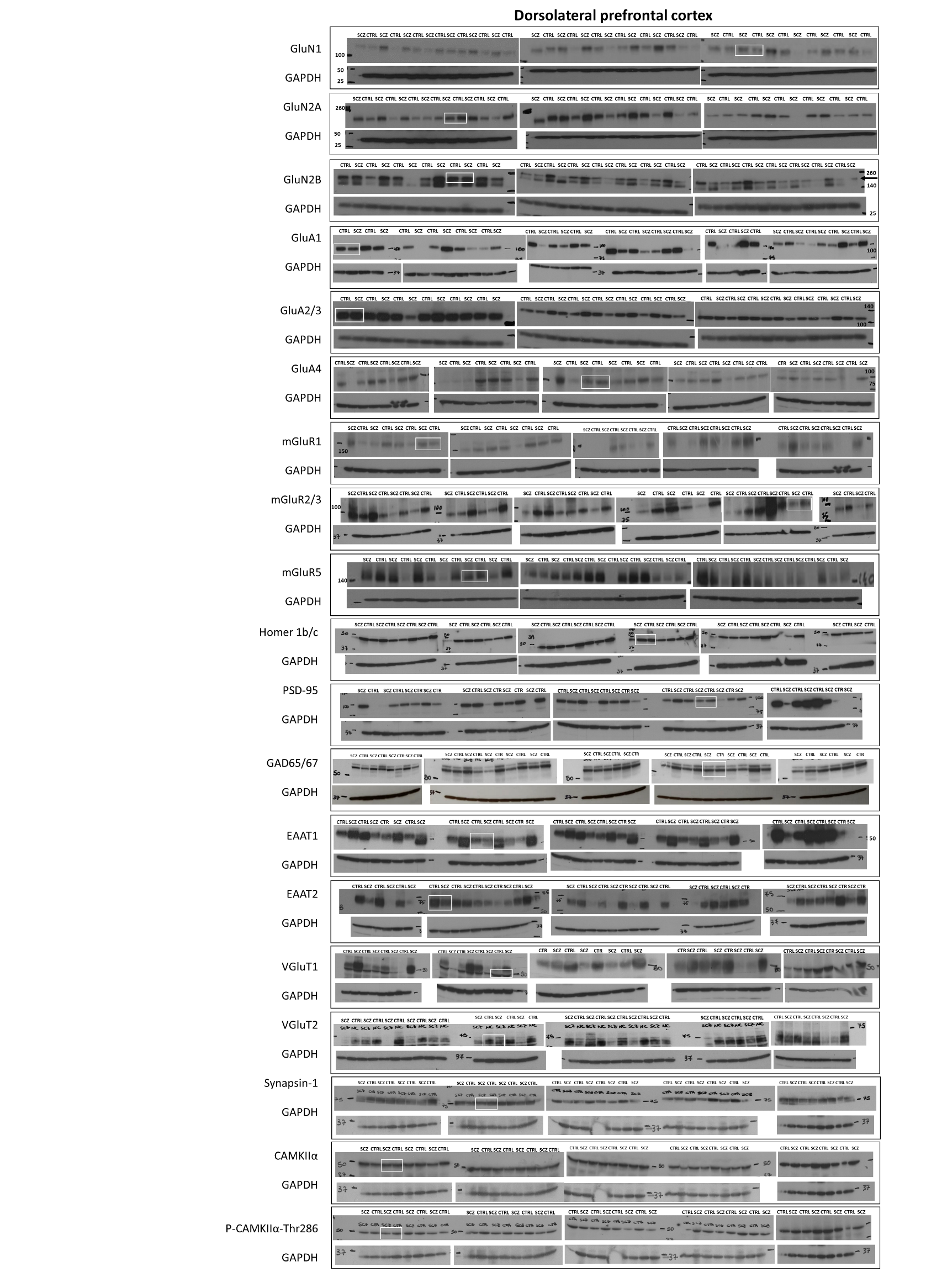


**Supplementary Figure 2.** Raw blots of figure 3a’. Abbreviations: CTRL = Control; SCZ = schizophrenia.


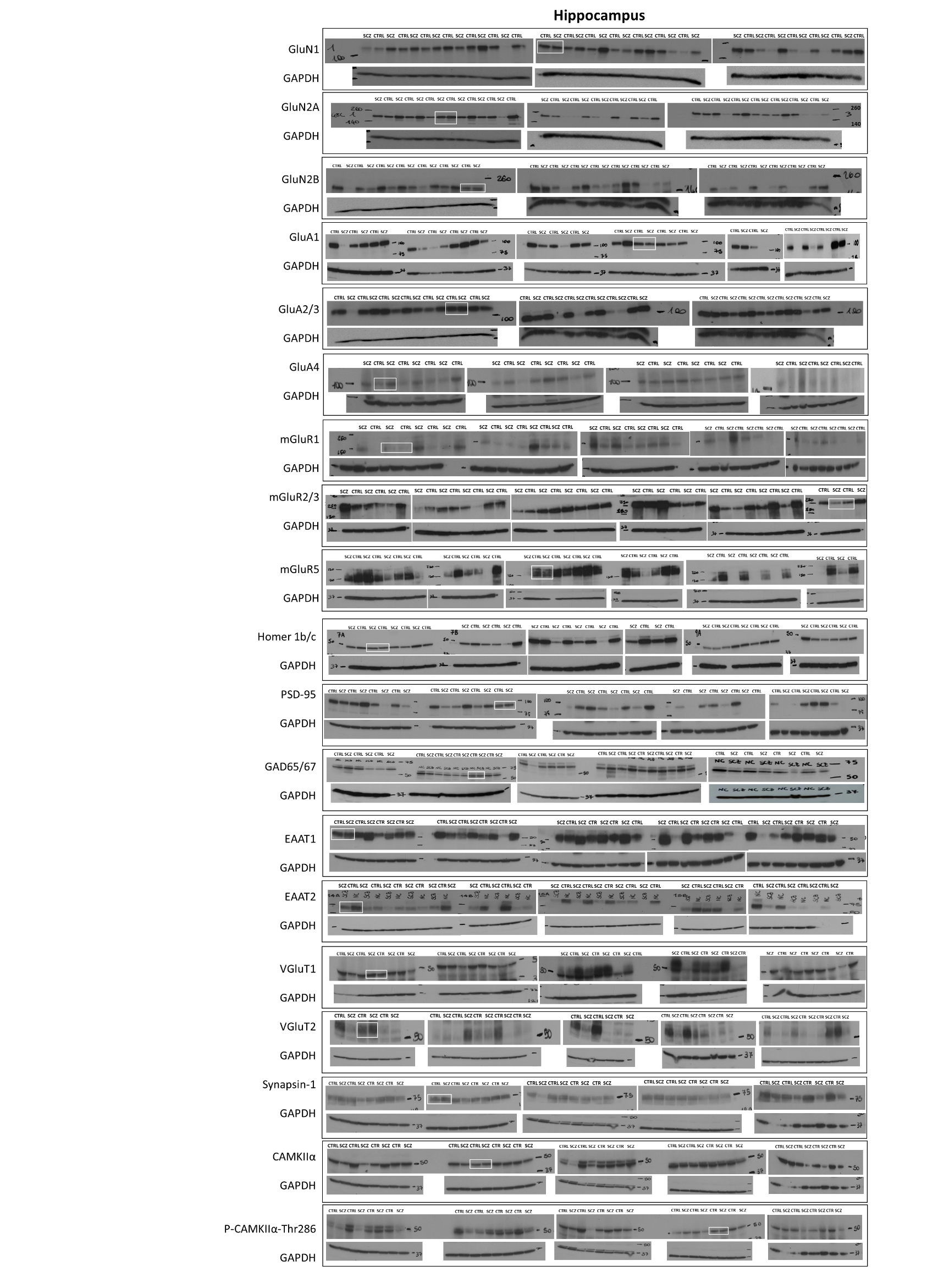


**Supplementary Table 7.** Out of Bag Brier Scores (i.e. prediction errors) achieved by the iterative Random Forest (iRF) with 100’000 trees at different tuning parameters (i.e. number of iterations and regularization factors) in the post-mortem dorsolateral prefrontal cortex (DLPFC) and hippocampus (HIP), respectively. The lower Brier Scores, the better iRF fitting to the OOB data

|  |  | **Iterations** | | | | | | | | | |
| --- | --- | --- | --- | --- | --- | --- | --- | --- | --- | --- | --- |
|  | **Reg.Fact^#^** | **1** | **2** | **3** | **4** | **5** | **6** | **7** | **8** | **9** | **10** |
| **DLPFC** | 1.0 | 0.2174 | 0.1937 | 0.1897 | 0.1890 | 0.1882 | 0.1872 | 0.1868 | 0.1861 | 0.1858 | 0.1856 |
|  | 0.9 | 0.2174 | 0.1937 | 0.1897 | 0.1891 | 0.1881 | 0.1871 | 0.1870 | 0.1862 | 0.1856 | 0.1857 |
|  | 0.8 | 0.2173 | 0.1936 | 0.1892 | 0.1885 | 0.1882 | 0.1871 | 0.1863 | 0.1858 | 0.1858 | **0.1855*** |
| **HIP** | 1.0 | 0.2248 | 0.1824 | 0.1750 | 0.1707 | 0.1683 | 0.1667 | 0.1660 | 0.1660 | 0.1658 | 0.1658 |
|  | 0.9 | 0.2248 | 0.1824 | 0.1748 | 0.1704 | 0.1682 | 0.1671 | 0.1660 | 0.1659 | 0.1657 | 0.1657 |
|  | 0.8 | 0.2246 | 0.1817 | 0.1742 | 0.1697 | 0.1674 | 0.1664 | **0.1654*** | 0.1656 | 0.1655 | 0.1657 |

**Abbreviations:** DLPFC: Dorsolateral prefrontal cortex; HIP: Hippocampus; Reg.Fact: Regularization Factor;

^#^For regularization factor ≤ 0.7, almost all the variables have zero weights just at the first iteration. Consequently, no sufficient variables can be selected for the building of each tree to be included in the iRF; *Optimal fitting (i.e. the lowest OOB Brier Score), achieved by the iRF with 100’000 trees, was found for iRF with 10 and 7 iterations and regularization factor at 0.8 for DLPFC and HIP data, respectively.

**Supplementary Figure 3.** Out of Bag Brier Score (i.e. prediction error) with respect to the increasing number of trees in the iterative Random Forest at varying number of iterations and regularization factors in the post-mortem dorsolateral prefrontal cortex (DLPFC) and hippocampus (HIP), respectively


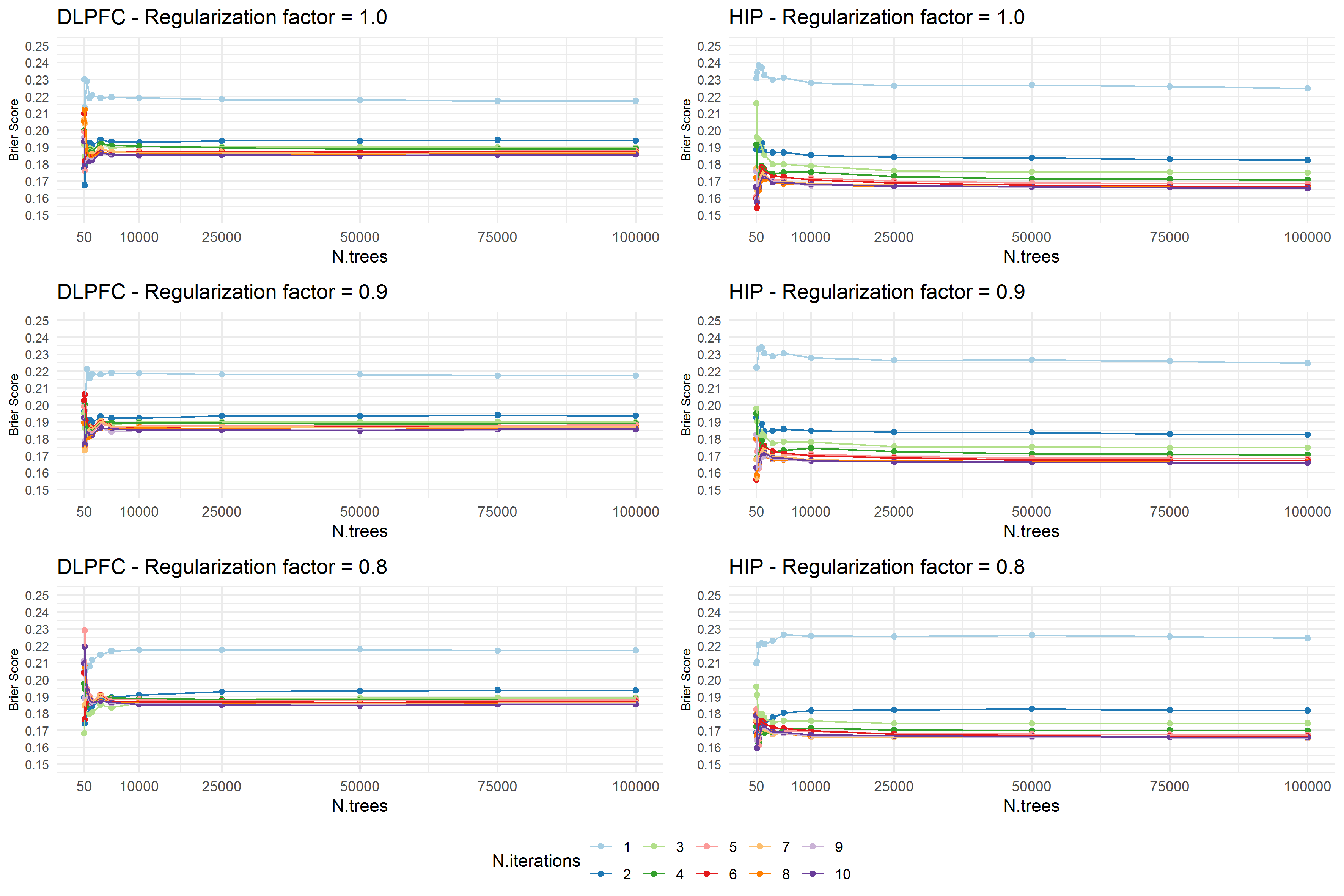


Panels on the left side are referred to results from iRF using DLPFC data whereas panels on the right side are referred to the ones using HIP data. As shown, Brier Score is drastically reduced just passing from the first to the second iteration, independently by the number of trees or regularization factors. Furthermore, higher volatility between Brier scores is detected at lower number of trees (less than 10’000 trees) and gradually reduces with the increasing number of trees. Brier scores achieve lower values in a iRF with at least two iterations and a regularization factor of 0.8.

**Supplementary Figure 4.** Classification And Regression Tree (CART) modeling via Recursive Partitioning And Regression Tree (RPART) analysis


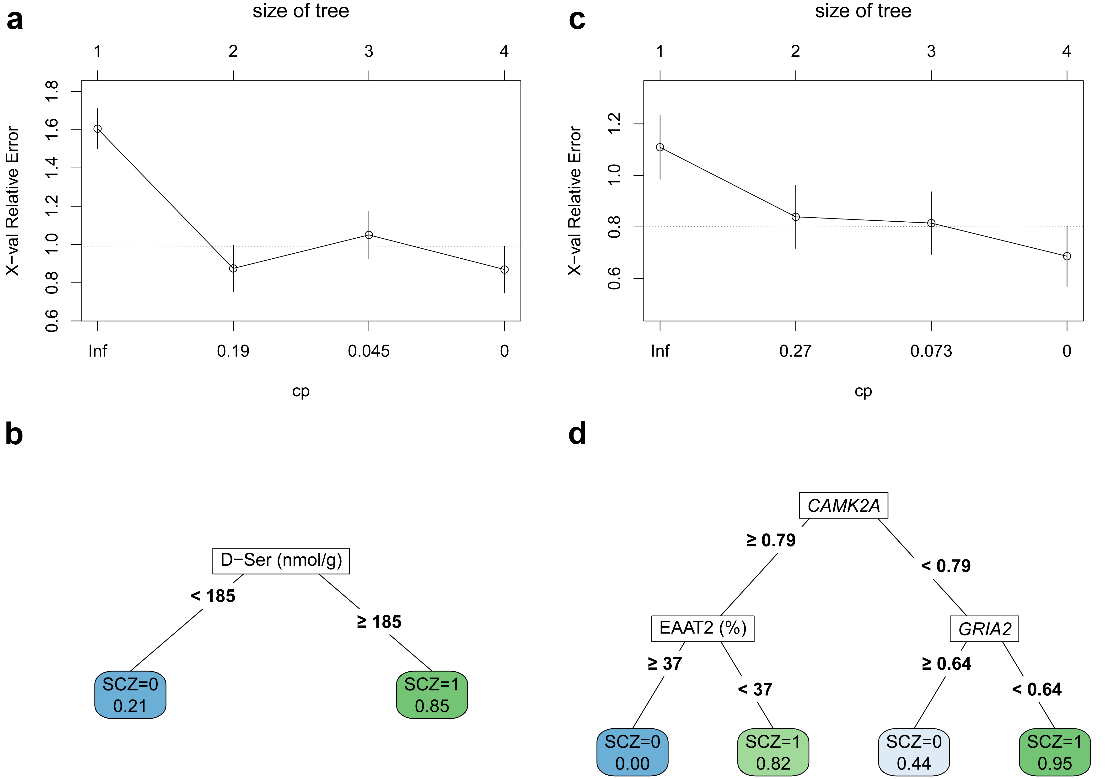


RPART identifies subjects subgroups at different probability of having the schizophrenia (SCZ) with the building of a decision tree (CART) using the post-mortem dorsolateral prefrontal cortex (DLPFC) (**a, b**) and hippocampus (HIP) (**c, d**) data, respectively. The optimal size of each tree (i.e. the number of terminal nodes in each tree) was established by looking at the 10-fold cross-validated prediction error achieved for each possible tree size (i.e. the “X-val Relative Error” that correspond to geometric means of the cross-validated errors along with ± 1 standard error (SE) reported as error bars) and the one for which leftmost prediction error value lies below 1 SE above the minimum value (i.e. the horizontal dashed line) was considered. For each considered tree, observations (i.e. subjects) are reweighted by the Inverse Probability Weights (IPW); at least 3 (weighted) observations must exist in a node for a split and at least 6 (weighted) observations must exist in any terminal node. The complexity parameter (“cp”) allows the user to prune the full tree to the desired size. The optimal tree sizes are 2 and 4 (terminal nodes) for DLPFC (**a**) and HIP (**c**) data, respectively. The tree-growing algorithm recursively splits the data into subgroups, choosing the best binary split for each considered variable at issue, to identify the most homogeneous sets within each node and the most heterogeneous ones between the nodes. Splitting variables are shown between branches, while condition sending subjects to left or right sibling is on relative branch. Squares represent subgroups of subjects whereas circles represent the terminal nodes. Numbers inside circles represent the probability of having the disease (from 0 to 1; bottom) and whether the subjects have or not the disease (top), respectively. The latter information was deduced on the basis of predicted probability, using 0.5 as the reference threshold. For DLPFC data, subjects with D-serine (D-Ser) ≥ 185 nmol/g achieve 85% chance of having the SCZ whereas those with D-Ser < 185 nmol/g are only 21% more likely to have the disease (**b**). For HIP data, subjects with *CAMK2A* expression < 0.79 and (at the same time) with *GRIA2* expression < 0.64 achieve 95% chance of having the SCZ whereas those with *CAMK2A* expression ≥ 0.79 and EAAT2 ≥ 37 % are more likely to do not have the disease at all (probability of 0%) (**d**). The discriminatory accuracy achieved by both CARTs and assessed by computing the Area Under the ROC Curve (AUC) was AUC=0.73 (95%CI: 0.60-0.85) and AUC=0.92 (95%CI: 0.83-0.99) for DLPFC and HIP data, respectively.

**Supplementary References**

1 van Buuren, S. & Groothuis-Oudshoorn, K. MICE: Multivariate Imputation by Chained Equations in R. *J Stat Softw* **45**, 1-67 (2011).
